# Are wheezing, asthma and eczema in children associated with mother’s health during pregnancy? Evidence from an Australian birth cohort

**DOI:** 10.1186/s13690-021-00718-w

**Published:** 2021-11-09

**Authors:** Kabir Ahmad, Enamul Kabir, Gail M. Ormsby, Rasheda Khanam

**Affiliations:** 1 School of Business, Faculty of Business, Education, Law and Arts, and Centre for Health Research, University of Southern Queensland, Toowoomba, Australia; 2 Research Unit, Purple Informatics, Dhaka, Bangladesh; 3 Present Address: School of Business, Faculty of Business, Education, Law and Arts, and Centre for Health Research, University of Southern Queensland, Toowoomba, Australia; 4 School of Sciences, Faculty of Health, Engineering and Sciences, and Centre for Health Research, University of Southern Queensland, Toowoomba, Australia; 5 Independent Researcher, Professional Studies, Faculty of Business, Education, Law and Arts, University of Southern Queensland, Toowoomba, Australia

**Keywords:** Children’s respiratory disease, Allergic disease, Wheezing, Asthma, Eczema, Maternal health in pregnancy, Maternal medications, Maternal body mass index, Maternal smoking

## Abstract

**Background:**

This study investigated the prevalence of wheezing, asthma, and eczema among Australian children using longitudinal data from birth to 15 years of age. This study also examined the association between maternal health status during pregnancy and their offspring’s respiratory and allergic morbidities using sex-segregated data.

**Methods:**

This study used data from the Longitudinal Study of Australian Children (LSAC) where approximately 5000 children of a birth cohort across Australia were surveyed in 2004. These children were followed biennially in eight waves up to their age of 15 years until 2018. The status of the children’s wheezing, asthma, and eczema were reported by the mothers upon doctors’ diagnosis (for asthma) or self-assessment (for wheezing or eczema). Binomial logistic regression models were used to analyse associations between maternal health during pregnancy and their children’s health outcomes.

**Results:**

Asthma prevalence among 0–1-year aged children was 11.7%, increased to 15.4% when the children were 10–11 years old, and then decreased to 13.6% when they were 14–15 years old. Wheezing and eczema were most prevalent when the children were 2–3 years old (26.0 and 17.8% respectively) and were least prevalent when the children were 14–15 years old (7.3 and 9.5% respectively). Maternal asthma, smoking during pregnancy, and pre-pregnancy obesity were significantly associated with an increased risk of wheezing and asthma in Australian children. Childhood eczema was associated only with maternal asthma. These associations were stronger among male children up to age 10–11 and during adolescence (12–15 years of age), female children were more prone to wheezing, asthma, and eczema.

**Conclusion:**

This is a comprehensive longitudinal study of Australian children (0–15 years of age) to assess the prevalence (with sex-specific differences) of wheezing, asthma and eczema as well as the association between these respiratory and allergic morbidities and maternal health during pregnancy. The study findings suggest that careful medical and obstetric monitoring, improved specific age-sex wise risk factor prevention for children and health promotion for pregnant women would help protect child health.

**Supplementary Information:**

The online version contains supplementary material available at 10.1186/s13690-021-00718-w.

## Background

Childhood respiratory and allergic diseases, wheezing, asthma, and eczema, are leading causes of global morbidity [1]. The 2000–2003 International Study of Asthma and Allergies in Childhood found that 14.1 and 7.3% of children aged 13–14 years were currently suffering from asthma or eczema, respectively [1]. The 2018 Australian health survey revealed that among children aged 5–14 years, 11% reported current asthma, making asthma the leading health burden in that age group [2]. A longitudinal study conducted in 2009 found that 16.9% of Australian children, born in 2004, experienced wheezing or asthma within the first 3 years of life [3]. Wheezing, asthma and eczema [4] pose significant long-term health burdens to children, such as poor lung function or development of persistent asthma in later life [5–8]. Furthermore, the incidences of wheezing, asthma or eczema are influenced by maternal health and environmental conditions [9–11] which include maternal exposures to asthma, obesity, antibiotic/antidepressant medication use or smoking during pregnancy [4, 8, 12–16]. Therefore, comprehensive research related to longitudinal prevalence of wheezing, asthma, and eczema among children, taking maternal health during pregnancy into account, is a public health priority.

As a definition, wheezing has been defined as a “continuous high-pitched sound with musical quality emitting from the chest during expiration” and results in “the narrowing of intrathoracic airways and expiratory flow limitation” [17]. Some studies have shown that approximately 25% of children with persistent asthma had wheezing symptoms in their early life [17–19]. The Global Initiative for Asthma (GINA) states that “asthma is a syndrome with a highly variable clinical spectrum, characterised by airway inflammation” [17].

Asthma also causes shortness of breath and chest tightness, and can cause cough [5]. The definition of eczema is based on the Hanifin and Rajka validated criteria [20], which include: itchy skin conditions in the past 12 months, history of skin creases, history of dry skin in the past 12 months and visible flexural dermatitis. Though there are several cross-sectional studies [21], the prevalence of each of these conditions (wheezing, asthma and eczema) are understudied in contemporary population-based longitudinal studies of children’s health.

Pregnancy is a crucial period in determining the future health of the offspring [10] and hence, further understanding on mother’s pregnancy health and children’s respiratory and allergic diseases are necessary. To date several studies have shown that maternal asthma during pregnancy, pre-pregnancy obesity, and gestational weight gain are associated with an increased risk of asthma or wheezing in the offspring [12, 22–24]. However, most of these existing studies followed children from the first year of life to preschool or early school age [12, 25]. There is little longitudinal information about associations between maternal health during pregnancy and long-term respiratory or allergic health outcomes in children through following the participants up from birth to adolescence [8, 26].

A growing body of literature reveals that maternal risk behaviours, such as smoking during pregnancy [27] and maternal use of some medications [28, 29], are associated with increased risk of wheezing or asthma in the offspring. Although prenatal or postnatal smoking is a significant risk factor for wheezing and asthma among infants and preschool-age children [27, 30], it is not well known how the number of cigarettes smoked during pregnancy effects the association. Some studies have shown that maternal use of medications, which includes paracetamol use [31] or high doses of folic acid [29] during pregnancy had influenced health outcomes in children such as increased risk of childhood asthma [14, 32]. However, few studies have examined the effects of anti-depressant or antibiotic medication use during pregnancy on childhood wheezing, asthma or eczema [31, 33]. In Canada, the Manitoba province population-based study from 1996 to 2012, revealed that prenatal antibiotic exposure was associated with an increased risk of asthma [33]. No population based comprehensive study examined the effects of anti-depressant or antibiotic medication use during pregnancy on Australian children.

Sex dimorphism has long been recognised to childhood morbidities, but few studies investigated the sex-specific differences on children’s respiratory and allergic diseases [34, 35]. For example, a 2003–2008 study in Greece showed that male:female ratio of current and lifetime wheezing and asthma increased; although, irrespective of sex, asthma diagnosis declined among school-age children, but not among preschool wheezers [34]. However, these studies only focused on prevalence and lacked investigation on the sex-specific associations of familial heredity or maternal health during pregnancy. Few studies have used a single population-based prospective study on both respiratory and allergic diseases [36, 37], or adjusted for confounding factors of maternal health during pregnancy to determine the age and sex-specific effects on children’s respiratory and allergic diseases [14, 25]. Longitudinal investigation of sex differences of wheezing, asthma and eczema in Australian children (birth to 15 years of age), associated with maternal health during pregnancy, is limited.

The present study, therefore, aims to determine the sex-specific longitudinal prevalence of respiratory and allergic diseases, wheezing, asthma, and eczema, among children from birth to the age of 15 years on a population based longitudinal data of Australian children. Further, it investigates the association between maternal health or health risk behaviours (asthma, gestational age, maternal pre-pregnancy BMI, maternal smoking or use of antibiotics or antidepressants) during pregnancy and the offspring’s wheezing, asthma, and eczema throughout childhood (including sex-disaggregated differences) to the age of 15 years. Findings of this study would broaden the understanding of the age and sex specific long-term aetiology of childhood respiratory and allergic morbidities.

## Methods

### Data source and sample selection

The data was obtained from eight waves of the 2004–2018 Longitudinal Study of Australian Children (LSAC). LSAC is a representative household survey of Australian children that biennially collects information on the health (physical and socio-emotional), and learning development of Australian children based on the context of the bio-ecological framework of human development [38]. The LSAC data is collected from the parents or caregivers of the children of participating households and from the children themselves (from age 12 onward), through self-completed questionnaires or face-to-face interviews with trained interviewers. A multi-stage sampling technique was used to select the LSAC respondents. The household is the primary sampling unit. Further details regarding LSAC survey design and methodology can be found elsewhere [39].

The LSAC dataset contained information on children’s wheezing, asthma, and eczema-related health and their mothers’ self-reported health (asthma, pre-pregnancy obesity) and health-risk behaviours (medications, smoking habits) during pregnancy from the biological mother-child pair as well as eight waves of the child’s ongoing health up to 15 years of age.

After excluding children from non-biological parents, the final sample was 4977 in Wave 1. There were drop-outs in the subsequent waves and at Wave 8 the attrition rate was 38.8% sustaining 2960 mothers and children in the sample. An additional appendix file shows the total LSAC participants, the attrition rates and the final study sample after exclusion of non-mother parents for each of the waves in Fig. A1 (see Additional file 1). This figure also shows the loss to follow-up sample for Wave 2 to Wave 8 calculated from eligible sample of the first wave (baseline wave) to the particular wave.

### Outcome variables

The outcome variables of this study were whether the children: (i) had wheezing, (ii) were ever diagnosed with asthma, (iii) had current asthma for which they took medication, or (iv) had eczema. The LSAC survey respondents’ (parents/caregivers) were asked the following questions: (i) Has your child had an illness with wheezing in the chest which lasted for a week or more in the last 12 months?; (ii) Has a doctor ever told you that your child has asthma?; (iii) Has your child taken any medication for asthma in last 12 months?; and (iv) Does your child have any ongoing conditions with eczema? A binary variable was used to capture each of these responses (Yes = 1 and No = 0).

Existing literature shows that although a good portion of preschool children have wheezing, not all of them diagnosed with asthma when they reach school age [17, 19]. In LSAC, wheezing condition was monitored in every wave, however, asthma was identified if the respondents reported doctor diagnosed asthma and it was started to trace first when the children reached 2–3 years age. Hence, this study followed up both wheezing and asthma separately, though many international multi-country or national studies determined asthma prevalence by assessing presence of wheezing as a symptom of asthma [5]. We took the opportunity to separately identify wheezing and asthma of LSAC to increase the specificity of these respiratory diseases among children over time.

### Independent variables

The independent variables considered in this study were based on the existing literature on this topic [13, 23, 40, 41]. The following independent variables were used: (i) incidence of asthma during pregnancy, (ii) mother’s pre-pregnancy BMI, (iii) gestational age at birth, (iv) maternal smoking during pregnancy, (v) the number of cigarettes smoked by mother during pregnancy, and (vi) the use of antibiotics or antidepressant medications taken by mother during pregnancy.

The data for the variable maternal asthma come from this question: ‘During the pregnancy with child, did (you/child’s mother) take any medicines or tablets on a doctor’s prescription for asthma?’. If a mother used any medication for the treatment of asthma, then the response was categorised as Yes =1; if not, it was categorised as No = 0. Maternal BMI was calculated from their pre-pregnancy height and weight recorded in Wave 1. Mothers’ BMI was categorised into four groups according to World Health Organization (WHO) guidelines: (i) ‘underweight’ (BMI < 18.50), (ii) ‘healthy weight’ (18.5 ≤ BMI < 25), (iii) ‘overweight’ (25 ≤ BMI < 30), and (iv) 'obese' (BMI ≥ 30). Approximately 20% of respondents did not provide height or weight; therefore, these mothers were grouped into a ‘not measured’ category. The children’s gestational age was recorded in weeks and categorised into three categories: (i) on time (37–41 weeks), (ii) early (36 weeks or less), (iii) or late (42 weeks or more). Information on maternal smoking during pregnancy was collected in Wave-1 of LSAC surveys. If mothers smoked during their pregnancy, the number of cigarettes smoked during the first trimester of pregnancy was recorded in the study. From this record, this study categorised data as follows: (i) none, (ii) < 10 daily, and (iii) 11+ daily. The information on medication use during pregnancy was also collected via mothers’ responses to ‘What prescribed medicines or tablets were taken during pregnancy?’. If mothers took any medications related with antibiotics or antidepressants, the responses were coded with dichotomous values where ‘Yes’ = 1 and ‘No’ = 0 for each of these two types of medications.

### Control variables

Based on existing literature [3, 13, 14, 32], this study considered the following confounding variables. Socio-demographic covariates included were (i) age of the mother (<=18, 19–34, > = 35 years), (ii) gender of the child (male or female), (iii) whether English is spoken at home (yes or no), (iv) whether mother is married with a partner, with a de facto partner, or single, (vi) indigenous status (yes or no), (vii) education of the mother (year 12 or less, professional qualification, graduate diploma, or postgraduate), (viii) family income (five quantiles), and (ix) remoteness of the family residence (highly accessible, accessible/moderately accessible, or remote/very remote). Other health or health-behaviour related confounders considered were (i) the type of birth (normal, caesarean, or other), (ii) birthweight (<=2500 g or > 2500 g), (iii) the immunisation status of the child (completely up to date or not), and (iv) the mother’s quality of sleep in the year prior to childbirth (very good/fairly good, fairly bad or very bad). The home condition related one relevant confounder was (i) home exterior condition (fair/well-kept exterior, bad/poor Exterior or not sighted).

### Statistical analysis

Descriptive statistics of the characteristics of the eight LSAC waves of sampled children and mothers have been presented using weighted frequency (n) and percentages (%). Further, descriptive analysis of the characteristics of the loss to follow-up samples on the outcome and explanatory variables have been performed. We performed this analysis to assess whether there were any bias on the loss of the sampled subjects. Multivariate analyses of binomial logistic regressions were employed to investigate the associations between maternal asthma, maternal obesity, gestation age, smoking and medication use (antibiotics or antidepressants) with the offspring’s risk of exposure to childhood wheezing, asthma and eczema. All of the maternal illness and health risk exposure variables were measured during the pregnancy, with the exception of obesity, for which the mothers reported their pre-pregnancy height and weight. This study also investigated these associations with sex-segregated data. For ease of interpretation, results of the multivariate analyses of the binomial logistic regressions are presented in the form of odds ratios (OR) with 95% confidence intervals (CI). A *p*-value of 0.05 or lower was considered statistically significant. All statistical analyses were performed using Stata (release 15) statistical software.

### Loss to follow-up

**Fig. 1:**
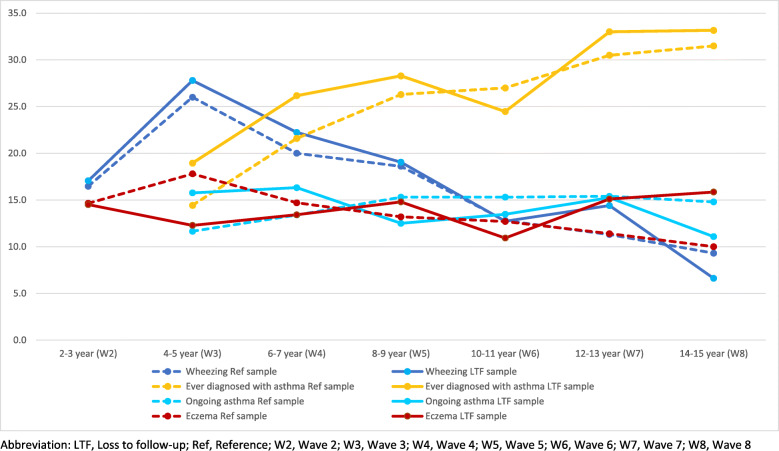
The prevalence of wheezing, asthma and eczema among the children of loss to follow-up sample compared to the reference wave’s whole sample

Figure 1 showed the comparison of the prevalence of wheezing, asthma and eczema among the children who were lost in the follow-up wave compared to the whole sample of that wave. The results of the analysis showed minor differences in the prevalence of loss to follow-up sample of each wave compared to the whole sample of the that wave. Moreover, the trends of the prevalence for each of the diseases were almost similar in the loss to follow-up sample. In addition to Fig. 1, we have presented a detailed picture of this outcome variables’ analysis in Table A1 of Additional file 1. Further, we have anlysed the frequency and percentages of the independent variables of this study on the loss to follow-up sample and it has been shown in Table A2 of the Additional file 1. The baseline characteristics of the loss to follow-up sample of each wave were similar to the characteristics of whole baseline sample regarding the explanatory variables. So, we may expect that there are no bias in the estimates of the statistical models as we observed no bias in the loss to follow-up samples over time.

## Results

### Prevalence of respiratory and allergic morbidities

**Fig. 2:**
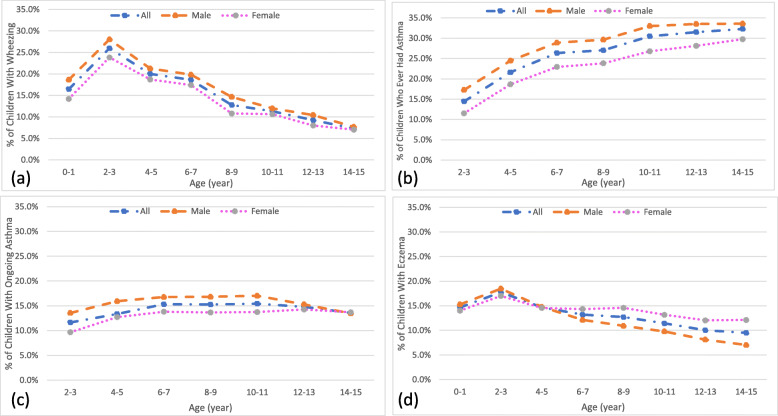
The percentage of Australian children, by age and sex, who (a) experienced wheezing in the last 12 months, (b) ever had diagnosed with asthma, (c) experienced ongoing asthma, and (d) experienced eczema in the last 12 months. The values of data points are shown in an additional appendix (see Additional file 2)

The prevalence of wheezing among children in this study in their first year of life was 16.5% and increased to 26.0% when the children were aged 2–3 years (Fig. 2). However, in every subsequent follow-up, there was a gradual decrease, and the prevalence of wheezing dropped to 7.3% by the children’s 14-15th year. The prevalence of asthma among children 2–3 years old was 11.7%. It increased to 15.3% between 6 and 10 years but then went down to 13.6% among adolescents aged 14–15 years. Prevalence of current asthma was slightly higher among male children up to the age of 12–13 years; however, increased prevalence was observed among female children aged 14–15 years. The prevalence of eczema was 14.7% among children aged 0–1, and it decreased to 9.5% by the time children reached adolescence. Eczema prevalence was higher among male children until age 4–5 years but increased among female children until it was 12.1% among females aged 14–15 years, compared to 7.0% among males in the same age group (Fig. 2).

### Maternal health

In Wave 1, among the mothers, 7.2% had asthma during their pregnancy, 14.4% were obese, and 15.1% smoked during the pregnancy. Furthermore, 4.4% of mothers smoked 11 cigarettes (or more) daily in their 1st trimester of pregnancy, 10.5% took antibiotic medication, and 2.1% took antidepressant medication during the pregnancy period (Table 1).

**Table 1:** Sample characteristics across the LSAC Waves

VARIABLES	Age 0–1	Age 2–3	Age 4–5	Age 6–7	Age 8–9	Age 10–11	Age 12–13	Age 14–15
	n (%)	n (%)	n (%)	n (%)	n (%)	n (%)	n (%)	n (%)
**N**	4977	4485	4264	4088	3922	3574	3097	2960
**EXPOSURE VARIABLES**								
**Mother had asthma during pregnancy**								
*No*	4619 (92.81)	4166 (92.89)	3960 (92.87)	3799 (92.93)	3633 (92.63)	3310 (92.61)	2862 (92.41)	2735 (92.40)
*Yes*	358 (7.19)	319 (7.11)	304 (7.13)	289 (7.07)	289 (7.37)	264 (7.39)	235 (7.59)	225 (7.60)
**Pre-pregnancy Obesity of Mother**								
*Underweight*	469 (9.42)	429 (9.57)	403 (9.45)	385 (9.42)	367 (9.36)	339 (9.49)	283 (9.14)	271 (9.16)
*Healthy weight*	1786 (35.89)	1675 (37.35)	1617 (37.92)	1567 (38.33)	1516 (38.65)	1395 (39.03)	1249 (40.33)	1191 (40.24)
*Overweight*	1007 (20.23)	944 (21.05)	917 (21.51)	877 (21.45)	837 (21.34)	782 (21.88)	682 (22.02)	648 (21.89)
*Obesity*	714 (14.35)	674 (15.03)	642 (15.06)	619 (15.14)	602 (15.35)	550 (15.39)	483 (15.6)	459 (15.51)
*Not measured*	1001 (20.11)	763 (17.01)	685 (16.06)	640 (15.66)	600 (15.3)	508 (14.21)	400 (12.92)	391 (13.21)
**Gestational age at birth**								
*On time (37–41 weeks)*	4407 (88.55)	3977 (88.67)	3786 (88.80)	3620 (88.54)	3492 (89.03)	3197 (89.46)	2782 (89.82)	2641 (89.22)
*Early (36 weeks or less)*	338 (6.80)	302 (6.73)	290 (6.80)	279 (6.83)	250 (6.37)	220 (6.15)	190 (6.13)	179 (6.04)
*Late (42 weeks or more)*	232 (4.65)	206 (4.60)	188 (4.40)	189 (4.63)	180 (4.60)	157 (4.39)	125 (4.04)	140 (4.74)
**Mother ever smoked during pregnancy**								
*No*	4224 (84.88)	3797 (84.67)	3616 (84.81)	3451 (84.43)	3368 (85.87)	3063 (85.72)	2655 (85.72)	2549 (86.11)
*Yes*	753 (15.12)	688 (15.33)	648 (15.19)	637 (15.57)	554 (14.13)	511 (14.28)	442 (14.28)	411 (13.89)
**Mother’s smoking during 1st trimester**								
*None*	4347 (87.35)	3906 (87.08)	3724 (87.33)	3560 (87.09)	3463 (88.3)	3149 (88.11)	2721 (87.84)	2609 (88.13)
*< =10 cigarettes daily*	410 (8.23)	372 (8.29)	346 (8.11)	338 (8.26)	301 (7.68)	287 (8.03)	250 (8.09)	233 (7.86)
*11+ cigarettes daily*	220 (4.42)	207 (4.62)	194 (4.56)	190 (4.65)	157 (4.01)	138 (3.86)	126 (4.07)	119 (4.01)
**Antidepressant medication during pregnancy**								
*No*	4870 (97.86)	4385 (97.78)	4170 (97.79)	3989 (97.59)	3840 (97.92)	3501 (97.97)	3033 (97.93)	2919 (98.63)
*Yes*	107 (2.14)	100 (2.22)	94 (2.21)	99 (2.41)	82 (2.08)	73 (2.03)	64 (2.07)	41 (1.37)
**Antibiotic medication during pregnancy**								
*No*	4452 (89.46)	4009 (89.38)	3816 (89.5)	3658 (89.49)	3502 (89.28)	3202 (89.59)	2773 (89.53)	2642 (89.27)
*Yes*	525 (10.54)	476 (10.62)	448 (10.5)	430 (10.51)	420 (10.72)	372 (10.41)	324 (10.47)	318 (10.73)
**CONTROL VARIABLES**								
**Child Health Issues**								
**Birth weight**								
*Normal (2500–3999)*	4071 (81.79)	3677 (81.99)	3490 (81.84)	3343 (81.78)	3198 (81.53)	2896 (81.02)	2510 (81.04)	2400 (81.08)
*Low (< 2500)*	279 (5.61)	237 (5.29)	245 (5.75)	237 (5.79)	202 (5.15)	192 (5.38)	168 (5.42)	156 (5.27)
High (> = 4000)	627 (12.59)	571 (12.73)	529 (12.41)	508 (12.43)	522 (13.32)	486 (13.61)	419 (13.54)	404 (13.65)
**Immunisation status of children**								
*Up to date*	4516 (90.74)	4091 (91.21)	3885 (91.1)	3694 (90.36)	3557 (90.7)	3258 (91.16)	2841 (91.72)	2700 (91.21)
*Not up to date*	461 (9.26)	394 (8.79)	379 (8.9)	394 (9.64)	365 (9.3)	316 (8.84)	256 (8.28)	260 (8.79)
**Breastfed children up to 6 months**								
*Yes*	2328 (46.78)	2092 (46.65)	2008 (47.1)	1934 (47.31)	1891 (48.22)	1745 (48.83)	1551 (50.07)	1491 (50.39)
*No*	2649 (53.22)	2393 (53.35)	2256 (52.9)	2154 (52.69)	2031 (51.78)	1829 (51.17)	1546 (49.93)	1469 (49.61)
**Mother’s sleep quality in the year of childbirth**								
*Very good/Fairly good*	3478 (69.89)	3127 (69.72)	2966 (69.56)	2844 (69.57)	2736 (69.77)	2484 (69.5)	2149 (69.39)	2047 (69.16)
*Fairly bad*	1118 (22.46)	1000 (22.3)	961 (22.53)	921 (22.53)	899 (22.91)	817 (22.85)	715 (23.08)	685 (23.13)
*Very bad*	381 (7.66)	358 (7.98)	337 (7.91)	323 (7.9)	287 (7.32)	273 (7.65)	233 (7.53)	228 (7.71)

### Association of maternal health during pregnancy with offspring’s morbidities

Table 2 presents the associations between child wheezing and maternal health (asthma, BMI), risk factors (gestational age, smoking), and medication use (antibiotics, antidepressants) during pregnancy. Children of mothers who had asthma during pregnancy had an increased odds (OR: 1.5–2.5) of having wheezing until age 12–13. However, there was no significant association at the age of 14–15. Table 3 represents the associations between the above-mentioned maternal health and whether the children were ever diagnosed with asthma (cumulative effect). In all age groups at all the follow-ups, children’s odds of having been diagnosed with asthma (by a doctor or physician) were 2.5 times greater if their mother had asthma during the pregnancy. In all follow-up groups, the odds of experiencing current asthma were significantly higher (OR: 2.5–3.7) among children whose mothers had experienced asthma during their pregnancy compared to the children of mothers who did not have asthma (Table 4).

**Table 2:** The risk of experiencing wheezing among children based on the incidence of maternal asthma, other morbidities, and maternal health behaviours during pregnancy

Maternal health, risk factors, and medications during pregnancy	Age 0–1	Age 2–3	Age 4–5	Age 6–7	Age 8–9	Age 10–11	Age 12–13	Age 14–15
	OR (95% CI)	OR (95% CI)	OR (95% CI)	OR (95% CI)	OR (95% CI)	OR (95% CI)	OR (95% CI)	OR (95% CI)
	*N* = 4977	*N* = 4485	*N* = 4264	*N* = 4088	*N* = 3922	*N* = 3574	*N* = 3097	*N* = 2960
**Had asthma**								
*No (ref.)*								
*Yes*	1.49 (1.13–1.97)*	1.83 (1.4–2.4)**	1.47 (1.08–1.99)^Ϯ^	1.8 (1.3–2.49)**	1.91 (1.34–2.71)**	2.47 (1.69–3.61)**	1.69 (1.02–2.78)^Ϯ^	1.27 (0.72–2.24)
**Gestational age at birth**								
*On time (37–41 weeks, ref.)*								
*Early (36 weeks or less)*	1.37 (0.98–1.92)	1.54 (1.09–2.18)^Ϯ^	1.35 (0.91–2.01)	1.48 (0.97–2.26)	1.09 (0.66–1.78)	0.78 (0.38–1.57)	0.5 (0.21–1.19)	1.08 (0.49–2.39)
*Late (42 weeks or more)*	0.63 (0.41–0.98)^Ϯ^	1.00 (0.69–1.45)	1.53 (1.02–2.30)^Ϯ^	1.04 (0.67–1.63)	1.03 (0.63–1.70)	1.19 (0.67–2.1)	1.25 (0.66–2.38)	1.03 (0.50–2.1)
**Pre-pregnancy obesity**								
*Healthy weight (ref.)*								
*Underweight*	0.68 (0.48–0.96)^Ϯ^	0.89 (0.66–1.18)	0.87 (0.62–1.22)	0.77 (0.54–1.09)	0.85 (0.56–1.29)	0.91 (0.56–1.49)	1.21 (0.7–2.09)	0.60 (0.29–1.24)
*Overweight*	1.19 (0.94–1.51)	1.42 (1.15–1.74)**	1.12 (0.89–1.42)	1.02 (0.79–1.31)	1.22 (0.92–1.64)	1.1 (0.77–1.56)	1.47 (0.97–2.25)	1.28 (0.82–2.00)
*Obesity*	1.19 (0.91–1.56)	1.39 (1.1–1.75)*	1.31 (1.01–1.70)^Ϯ^	1.22 (0.93–1.61)	1.27 (0.92–1.75)	1.57 (1.1–2.23)^Ϯ^	1.41 (0.89–2.24)	1.27 (0.79–2.06)
*Not known*	1.50 (1.20–1.89)**	1.54 (1.23–1.93)**	1.36 (1.05–1.76)^Ϯ^	1.39 (1.06–1.82)^Ϯ^	1.21 (0.89–1.65)	1.39 (0.96–2)	1.82 (1.18–2.83)*	1.87 (1.17–2.99)*
**Smoking during 1st trimester**								
*None (ref.)*								
*< =10 cigarettes daily*	1.57 (1.17–2.1)*	1.52 (1.15–2.00)*	1.36 (0.99–1.86)	1.61 (1.16–2.25)*	1.36 (0.91–2.03)	1.03 (0.63–1.68)	1.3 (0.74–2.29)	1.18 (0.62–2.24)
*11+ cigarettes daily*	1.38 (0.95–2.00)	1.75 (1.21–2.52)*	1.8 (1.20–2.71)*	1.97 (1.28–3.01)*	1.54 (0.94–2.53)	1.56 (0.89–2.76)	1.11 (0.53–2.32)	2.61 (1.26–5.4)*
**Antibiotic medication**								
*No (ref.)*								
*Yes*	1.27 (0.99–1.62)	1.39 (1.1–1.77)*	1.48 (1.13–1.94)*	1.16 (0.87–1.55)	1.06 (0.76–1.47)	1.29 (0.88–1.88)	1.01 (0.61–1.67)	0.96 (0.54–1.71)
**Anti-depressant medication**								
*No (ref.)*								
*Yes*	1.29 (0.76–2.17)	1.92 (1.18–3.11)*	1.07 (0.60–1.93)	1.18 (0.65–2.11)	0.69 (0.31–1.53)	1.56 (0.76–3.2)	1.58 (0.62–4.01)	0.91 (0.27–3.03)

Notes: Ϯ *p* < 0.05 & > 0.01, * *p* < 0.01 & > 0.001, ** *p* < 0.001, the regression models were adjusted for several covariates outlined in ‘Control variables’ sub-section of ‘Methods’ section

**Table 3:** The risk of having ever been diagnosed with asthma among children based on the incidence of maternal asthma, other morbidities, and health behaviours during pregnancy (cumulative effect over age)

Maternal health, Risk factors, and medications during pregnancy	Age 2–3	Age 4–5	Age 6–7	Age 8–9	Age 10–11	Age 12–13	Age 14–15
	OR (95% CI)	OR (95% CI)	OR (95% CI)	OR (95% CI)	OR (95% CI)	OR (95% CI)	OR (95% CI)
	*N* = 4485	*N* = 4264	*N* = 4088	*N* = 3922	*N* = 3574	*N* = 3097	*N* = 2960
**Had Asthma**							
*No (ref.)*							
*Yes*	2.22 (1.65–3.00)**	2.34 (1.77–3.10)**	2.58 (1.94–3.44)**	2.42 (1.83–3.2)**	2.64 (1.96–3.56)**	2.9 (2.09–4.02)**	2.5 (1.78–3.52)**
**Gestational age at birth**							
*On time (37–41 weeks, ref.)*							
*Early (36 weeks or less)*	1.39 (0.88–2.18)	1.3 (0.88–1.91)	1.05 (0.71–1.55)	1.29 (0.88–1.89)	1.08 (0.71–1.64)	0.82 (0.50–1.35)	0.87 (0.54–1.41)
*Late (42 weeks or more)*	0.79 (0.49–1.28)	1 (0.66–1.52)	0.88 (0.59–1.32)	0.83 (0.56–1.24)	0.84 (0.55–1.28)	1.05 (0.66–1.67)	0.97 (0.62–1.52)
**Pre-pregnancy obesity**							
*Healthy weight (ref.)*							
*Underweight*	0.98 (0.68–1.4)	0.85 (0.61–1.17)	0.85 (0.63–1.16)	0.86 (0.64–1.16)	0.82 (0.60–1.12)	0.87 (0.62–1.21)	0.91 (0.65–1.28)
*Overweight*	1.37 (1.06–1.78)^Ϯ^	1.29 (1.03–1.62)^Ϯ^	1.27 (1.03–1.58)^Ϯ^	1.29 (1.04–1.59)^Ϯ^	1.27 (1.02–1.59)^Ϯ^	1.22 (0.96–1.56)	1.24 (0.97–1.58)
*Obesity*	1.54 (1.16–2.05)*	1.48 (1.15–1.89)*	1.39 (1.09–1.78)*	1.42 (1.12–1.80)*	1.41 (1.09–1.81)*	1.21 (0.92–1.59)	1.27 (0.97–1.67)
*Not known*	1.42 (1.08–1.88)^Ϯ^	1.33 (1.04–1.7)^Ϯ^	1.34 (1.05–1.71)^Ϯ^	1.36 (1.07–1.72)^Ϯ^	1.62 (1.26–2.09)**	1.65 (1.25–2.19)**	1.69 (1.26–2.25)**
**Smoking during 1st trimester**							
*None (ref.)*							
*< =10 cigarettes daily*	1.10 (0.78–1.54)	0.95 (0.69–1.30)	1.05 (0.77–1.43)	0.98 (0.72–1.35)	1.08 (0.77–1.51)	1.03 (0.71–1.5)	0.88 (0.59–1.32)
*11+ cigarettes daily*	1.67 (1.1–2.54)^Ϯ^	1.84 (1.24–2.72)*	1.66 (1.12–2.47)^Ϯ^	1.54 (1.01–2.34)^Ϯ^	1.6 (1.02–2.52)^Ϯ^	1.58 (0.97–2.57)	1.88 (1.14–3.09)^Ϯ^
**Antibiotic medication**							
*No (ref.)*							
*Yes*	1.20 (0.89–1.60)	1.09 (0.84–1.41)	1.19 (0.91–1.55)	1.15 (0.89–1.49)	1.18 (0.90–1.54)	1.01 (0.75–1.36)	1.02 (0.75–1.38)
**Anti-depressant medication**							
*No (ref.)*							
*Yes*	0.95 (0.49–1.85)	1.30 (0.74–2.29)	1.83 (1.07–3.12)^Ϯ^	1.27 (0.71–2.26)	1.49 (0.82–2.71)	1.3 (0.65–2.60)	0.50 (0.21–1.20)

Notes: Ϯ *p* < 0.05 & > 0.01, * *p* < 0.01 & > 0.001, ** *p* < 0.001; the regression models were adjusted for several covariates outlined in ‘Control variables’ sub-section of ‘Methods’ section. Abbreviation: *OR* Odds Ratio; ref., reference category

**Table 4:** The risk of having ongoing asthma among children based on the incidence of maternal asthma, other morbidities, and health behaviours during pregnancy

Maternal health, Risk factors, and medications during pregnancy	Age 2–3	Age 4–5	Age 6–7	Age 8–9	Age 10–11	Age 12–13	Age 14–15
	OR (95% CI)	OR (95% CI)	OR (95% CI)	OR (95% CI)	OR (95% CI)	OR (95% CI)	OR (95% CI)
	*N* = 4485	*N* = 4264	*N* = 4088	*N* = 3922	*N* = 3574	*N* = 3097	*N* = 2960
**Had asthma**							
*No (ref.)*							
*Yes*	2.53 (1.85–3.46)**	3.29 (2.47–4.40)**	3.32 (2.45–4.50)**	2.75 (2.03–3.73)**	3.74 (2.72–5.15)**	3.18 (2.22–4.56)**	2.63 (1.79–3.87)**
**Gestational age at birth**							
*On time (37–41 weeks, ref.)*							
*Early (36 weeks or less)*	1.26 (0.74–2.14)	1.23 (0.77–1.95)	1.00 (0.63–1.58)	0.81 (0.48–1.37)	0.63 (0.34–1.17)	0.65 (0.32–1.34)	0.72 (0.34–1.53)
*Late (42 weeks or more)*	0.83 (0.49–1.42)	1.03 (0.63–1.69)	1.21 (0.75–1.96)	0.99 (0.60–1.63)	1.30 (0.77–2.21)	1.11 (0.63–1.95)	1.30 (0.73–2.30)
**Pre-pregnancy obesity**							
*Healthy weight*							
*Underweight*	0.81 (0.53–1.23)	0.73 (0.48–1.1)	0.91 (0.62–1.32)	0.99 (0.69–1.44)	0.94 (0.62–1.42)	1.02 (0.66–1.58)	0.98 (0.61–1.55)
*Overweight*	1.37 (1.03–1.82)^Ϯ^	1.20 (0.92–1.56)	1.25 (0.96–1.63)	1.34 (1.03–1.74)^Ϯ^	1.3 (0.98–1.73)	1.37 (1.00–1.89)	1.18 (0.84–1.68)
*Obesity*	1.50 (1.10–2.06)^Ϯ^	1.39 (1.04–1.86)^Ϯ^	1.55 (1.15–2.09)*	1.40 (1.04–1.87)^Ϯ^	1.29 (0.94–1.78)	1.31 (0.92–1.86)	1.37 (0.96–1.96)
*Not known*	1.40 (1.04–1.89)^Ϯ^	1.23 (0.93–1.64)	1.46 (1.09–1.96)^Ϯ^	1.57 (1.18–2.09)*	1.78 (1.30–2.44)**	1.47 (1.03–2.11)^Ϯ^	1.41 (0.97–2.04)
**Smoking during 1st trimester**							
*None (ref.)*							
*< =10 cigarettes daily*	0.98 (0.66–1.44)	0.94 (0.64–1.38)	1.01 (0.68–1.52)	1.14 (0.77–1.7)	1.22 (0.77–1.91)	1.23 (0.76–1.99)	0.77 (0.44–1.36)
*11+ cigarettes daily*	1.53 (0.96–2.43)	1.91 (1.23–2.96)*	1.71 (1.08–2.71)^Ϯ^	1.40 (0.83–2.36)	1.78 (1.06–2.99)^Ϯ^	1.49 (0.84–2.65)	1.70 (0.89–3.25)
**Antibiotic medication**							
*No (ref.)*							
*Yes*	1.22 (0.89–1.68)	1.14 (0.85–1.54)	1.27 (0.95–1.72)	1.19 (0.88–1.61)	1.19 (0.86–1.64)	0.89 (0.61–1.31)	0.93 (0.62–1.39)
**Anti-depressant medication**							
*No (ref.)*							
*Yes*	0.95 (0.47–1.95)	1.33 (0.7–2.53)	1.53 (0.84–2.8)	0.86 (0.41–1.83)	1.48 (0.70–3.12)	1.43 (0.61–3.38)	0.90 (0.32–2.55)

Notes: Ϯ *p* < 0.05 & > 0.01, * *p* < 0.01 & > 0.001, ** *p* < 0.001, the regression models were adjusted for several covariates outlined in ‘Control variables’ sub-section of ‘Methods’ section. Abbreviation: *OR* Odds Ratio, ref., reference category

The odd of experiencing eczema was around 1.4 times higher among the children 0–1 and 4–5 years of age whose mother had experienced asthma in pregnancy compared to the offspring of non-asthmatic mothers (Table 5); this odds increased to 1.9 times when the children reached 6–7 years of age. However, from the age of 8–9 years until adolescence, the influence of maternal asthma during pregnancy on the odds of having eczema was insignificant (Table 5). Interestingly, maternal asthma during pregnancy had a greater effect on the odds of the offspring having asthma than on the odds of having eczema. This was the pattern across all age groups.

**Table 5:** The risk of experiencing eczema among children based on the incidence of maternal asthma, other morbidities, and health behaviours during pregnancy

Maternal health, Risk factors, and medications during pregnancy	Age 0–1	Age 2–3	Age 4–5	Age 6–7	Age 8–9	Age 10–11	Age 12–13	Age 14–15
	OR (95% CI)	OR (95% CI)	OR (95% CI)	OR (95% CI)	OR (95% CI)	OR (95% CI)	OR (95% CI)	OR (95% CI)
	*N* = 4977	*N* = 4485	*N* = 4264	*N* = 4088	*N* = 3922	*N* = 3574	*N* = 3097	*N* = 2960
**Had asthma**								
*No (ref.)*								
*Yes*	1.43 (1.06–1.94)^Ϯ^	1.2 (0.88–1.63)	1.45 (1.04–2.04)^Ϯ^	1.97 (1.40–2.77)**	1.38 (0.95–1.99)	1.56 (1.04–2.35)^Ϯ^	1.71 (1.09–2.67)^Ϯ^	1.48 (0.92–2.36)
**Gestational age at birth**								
*On time (37–41 weeks, ref.)*								
*Early (36 weeks or less)*	0.68 (0.44–1.05)	0.88 (0.57–1.34)	0.90 (0.56–1.46)	1.18 (0.71–1.95)	0.85 (0.45–1.62)	0.66 (0.31–1.44)	0.53 (0.22–1.26)	0.67 (0.28–1.62)
*Late (42 weeks or more)*	0.73 (0.47–1.14)	0.63 (0.4–1.01)	0.49 (0.27–0.89)^Ϯ^	1.03 (0.62–1.69)	0.97 (0.58–1.62)	0.63 (0.35–1.14)	0.53 (0.23–1.18)	1.00 (0.49–2.01)
**Pre-pregnancy obesity**								
*Healthy weight (ref.)*								
*Underweight*	0.98 (0.73–1.33)	0.67 (0.49–0.93)^Ϯ^	0.68 (0.48–0.97)^Ϯ^	0.76 (0.51–1.13)	0.73 (0.47–1.12)	1.14 (0.74–1.76)	0.92 (0.55–1.54)	0.82 (0.48–1.38)
*Overweight*	1.08 (0.86–1.36)	1.00 (0.80–1.25)	1.04 (0.81–1.34)	0.99 (0.75–1.3)	1.16 (0.88–1.53)	1.33 (0.98–1.82)	1.24 (0.86–1.77)	0.91 (0.62–1.33)
*Obesity*	1.06 (0.81–1.39)	1.14 (0.89–1.46)	1.17 (0.89–1.56)	1.32 (0.98–1.78)	1.27 (0.94–1.72)	1.24 (0.87–1.76)	1.23 (0.82–1.85)	1.77 (1.20–2.61)*
*Not known*	1.07 (0.84–1.36)	0.94 (0.73–1.2)	1.2 (0.91–1.59)	1.06 (0.77–1.46)	1.00 (0.72–1.4)	1.12 (0.77–1.65)	1.45 (0.95–2.20)	1.02 (0.65–1.61)
**Smoking during 1st trimester**								
*None (ref.)*								
*Occasional/< 10 daily*	0.95 (0.67–1.35)	1.18 (0.85–1.62)	1.16 (0.80–1.67)	0.83 (0.53–1.30)	0.84 (0.54–1.32)	0.81 (0.48–1.37)	0.63 (0.33–1.2)	0.56 (0.27–1.16)
*11+ daily*	0.71 (0.42–1.19)	0.92 (0.58–1.49)	0.76 (0.43–1.34)	1.42 (0.82–2.44)	0.84 (0.44–1.58)	0.70 (0.32–1.55)	1.09 (0.44–2.71)	1.12 (0.52–2.42)
**Antibiotic medication**								
*No (ref.)*								
*Yes*	0.95 (0.73–1.26)	1.03 (0.79–1.35)	1.03 (0.77–1.39)	1.30 (0.95–1.77)	1.29 (0.94–1.78)	1.19 (0.83–1.71)	1.14 (0.72–1.81)	1.35 (0.87–2.09)
**Anti-depressant medication**								
*No (ref.)*								
*Yes*	0.85 (0.47–1.54)	1.71 (0.97–2.99)	1.34 (0.72–2.48)	1.11 (0.56–2.21)	1.00 (0.49–2.05)	1.03 (0.47–2.28)	1.04 (0.37–2.91)	0.48 (0.13–1.75)

Notes: Ϯ *p* < 0.05 & > 0.01, * *p* < 0.01 & > 0.001, ** *p* < 0.001, the regression models were adjusted for several covariates outlined in ‘Control variables’ sub-section of ‘Methods’ section. Abbreviation: *OR* Odds Ratio; ref., reference category

The smoking status of mothers during pregnancy influenced their offspring’s health to varying extents across the respiratory and allergic morbidities. Children of mothers who smoked either less than 10 or 11 plus cigarettes during pregnancy showed 1.52 to 2.61 times greater odds to suffer from wheezing, although the odds ratio decreased after the age of seven years (Table 2). Children of mothers who smoked 11+ cigarettes a day during the first trimester showed higher odds to have ever been diagnosed with asthma (OR: 1.54–1.88) compared to the children of non-smoking mothers during pregnancy (Table 3). These children also showed higher odds to have been currently experiencing asthma until the age of 10–11 years (Table 4). However, maternal smoking during pregnancy, with any number of cigarettes, did not influence the odds of having eczema among the children at any of age groups (Table 5).

Maternal pre-pregnancy obesity had an influence on the offspring’s risk of experiencing wheezing and current or past asthma but not eczema (Tables 2, 3, 4 and 5). The odds of encountering wheezing illness was higher in children aged 2–5 of overweight and obese mothers compared to mothers of healthy weight, with odds ratio ranging from 1.31 to 1.42 (Table 2). The odds ratio of ever having been diagnosed with asthma ranged from 1.27 to 1.37 for children of overweight mothers and from 1.39 to 1.45 for children of obese mothers compared to the children of healthy weight mothers until age 10–11 (Table 3). A similar trend was present in the risk of currently having asthma among the children of overweight and obese mothers (Table 4).

If the mothers had taken antibiotics or antidepressant medication during pregnancy, their children showed higher odds of being afflicted with wheezing between ages 2 and 5 years than the children of mothers who did not take these medications. However, maternal medication use showed no association with the risk of children having asthma or eczema. Gestational age had a weak association with wheezing among preschool-aged children (0–5 years), but there was no association with asthma or eczema among the children at any age.

### Sex differences

This study also separately assessed the risks of and maternal associations with having respiratory or allergic morbidities among male and female children across all eight follow-ups. The statistical analyses were performed with sex-segregated data for all the outcome variables of this study with the same independent and control variables as shown in Table 2, 3 and 4. The detailed results of this sex-segregated analysis have been shown in Additional file 3. In Table 6, the compilation of odds of each of the disease outcomes (wheezing, ever diagnosed with asthma, ongoing asthma or eczema) of the children of mothers exposed to asthma during pregnancy, compared to the children of mothers who did not experience asthma during pregnancy, have been shown for all children and segregated by sex for the purpose of comparison. Up until the age of 10–11 years, male children had higher odds to encounter these morbidities than their female counterparts. However, as adolescents (12–15 years old), female children showed higher odds of having these morbidities (wheezing, ever had asthma, ongoing asthma or eczema) if their mother had asthma during pregnancy.

**Table 6:** Compilation of the odds of experiencing wheezing, having ever been diagnosed with asthma, having ongoing asthma, or having eczema among the children whose mothers experienced asthma during pregnancy, compared to the offspring of non-asthmatic mothers, for all children, male only and female only regression models of different ages

Respiratory or allergic morbidities	Age 0–1	Age 2–3	Age 4–5	Age 6–7	Age 8–9	Age 10–11	Age 12–13	Age 14–15
	OR (95% CI)	OR (95% CI)	OR (95% CI)	OR (95% CI)	OR (95% CI)	OR (95% CI)	OR (95% CI)	OR (95% CI)
	*N* = 4977	*N* = 4485	*N* = 4264	*N* = 4088	*N* = 3922	*N* = 3574	*N* = 3097	*N* = 2960
**Wheezing**								
***All children***								
*Wheezing = Yes*	1.49 (1.13–1.97)*	1.83 (1.4–2.4)**	1.47 (1.08–1.99)^Ϯ^	1.8 (1.3–2.49)**	1.91 (1.34–2.71)**	2.47 (1.69–3.61)**	1.69 (1.02–2.78)^Ϯ^	1.27 (0.72–2.24)
*Wheezing = No (ref.)*								
***Male only***								
*Wheezing = Yes*	*1.5(1.03–2.18)*^*Ϯ*^	1.93(1.34–2.76)**	1.77(1.20–2.63)*	2.07(1.35–3.18)**	1.97(1.22–3.17)*	3.28(2.01–5.34)**	1.56(0.78–3.11)	1.13(0.49–2.61)
*Wheezing = No (ref.)*								
***Female only***								
*Wheezing = Yes*	*1.46(0.94–2.24)*	1.73(1.14–2.65)*	1.15(0.70–1.91)	1.59(0.96–2.66)	1.8(1.03–3.15)^Ϯ^	1.72(0.92–3.21)	1.67 (0.81–3.46)	1.47 (0.69–3.15)
*Wheezing = No (ref.)*								
**Ever diagnosed asthma**								
***All children***								
*Ever had asthma = Yes*		2.22 (1.65–3)**	2.34 (1.77–3.10)**	2.58 (1.94–3.44)**	2.42 (1.83–3.20)**	2.64 (1.96–3.56)**	2.9 (2.09–4.02)**	2.5 (1.78–3.52)**
*Ever had asthma = No (ref.)*								
***Male only***								
*Ever had asthma = Yes*		2.76(1.86–4.11)**	2.75(1.86–4.07)**	2.69(1.81–3.98)**	2.87(1.93–4.28)**	3.04(1.98–4.67)**	2.66(1.69–4.18)**	2.35(1.46–3.79)**
*Ever had asthma = No (ref.)*								
***Female only***								
*Ever had asthma = Yes*		1.77(1.10–2.86)^Ϯ^	2.01(1.30–3.10)*	2.44(1.57–3.81)**	1.98(1.30–3.01)**	2.29(1.46–3.59)**	3.42(2.06–5.69)**	2.83(1.73–4.62)**
*Ever had asthma = No (ref.)*								
**Ongoing asthma**								
***All children***								
*Ongoing asthma = Yes*		2.53 (1.85–3.46)**	3.29 (2.47–4.40)**	3.32 (2.45–4.50)**	2.75 (2.03–3.73)**	3.74 (2.72–5.15)**	3.18 (2.22–4.56)**	2.63 (1.79–3.87)**
*Ongoing asthma = No (ref.)*								
***Male only***								
*Ongoing asthma = Yes*		3.17(2.10–4.77)**	4.09(2,76–6.08)**	3.30(2.17–5.00)**	2.74(1.79–4.20)**	3.66(2.36–5.68)**	3.17(1.93–5.21)	2.00(1.14–3.48)*
*Ongoing asthma = No (ref.)*								
***Female only***								
*Ongoing asthma = Yes*		2.08(1.27–3.41)*	2.68(1.69–4.24)**	3.40(2.14–5.39)**	2.83(1.81–4.43)**	4.09(2.55–6.56)**	3.38(1.94–5.86)**	3.30(1.88–5.82)**
*Ongoing asthma = No (ref.)*								
**Eczema**								
***All children***								
*Eczema = Yes*	1.43 (1.06–1.94)^Ϯ^	1.2 (0.88–1.63)	1.45 (1.04–2.04)^Ϯ^	1.97 (1.4–2.77)**	1.38 (0.95–1.99)	1.56 (1.04–2.35)^Ϯ^	1.71 (1.09–2.67)^Ϯ^	1.09 (2.67–0.018)
*Eczema = No (ref.)*								
***Male only***								
*Eczema = Yes*	1.46(0.96–2.21)^Ϯ^	1.13(0.73–1.75)	1.33(0.83–2.12)	2.08(1.29–3.36)*	1.76(1.05–2.96)^Ϯ^	2.18(1.24–3.84)*	1.16(0.58–2.34)	0.93(0.41–2.09)
*Eczema = No (ref.)*								
***Female only***								
*Eczema = Yes*	1.39(0.90–2.15)	1.28(0.82–2.01)	1.62(0.99–2.66)	1.88(1.15–3.07)	1.11(0.67–1.85)	1.03(0.58–1.83)	2.15(1.21–3.83)	1.95(1.08–3.51)
*Eczema = No (ref.)*								

Notes: Ϯ *p* < 0.05 & > 0.01, * *p* < 0.01 & > 0.001, ** *p* < 0.001, the regression models were adjusted for covariates outlined in ‘Control variables’ sub-section of ‘Methods’ section. Abbreviation: *OR* Odds Ratio; ref., reference category

## Discussion

This study comprehensively investigated the prevalence of respiratory and allergic morbidities (wheezing, asthma, and eczema) among children (birth to adolescence) and their association with maternal health factors and exposures during pregnancy (asthma, smoking, medication use, and pre-pregnancy obesity) using the LSAC data. The longitudinal prevalence of asthma in children aged 0–15 years of age measured in the eight biennial LSAC surveys (2004–2018) was 2–4 percentage points higher than the prevalence (11%) measured by the national health survey of Australian children aged 5–14 in 2018 [2].

Consistent with earlier studies [12, 14, 23, 27, 40, 42], this study also found that maternal asthma during pregnancy, smoking during pregnancy, and pre-pregnancy obesity were significantly associated with increased risks of wheezing and asthma among Australian children. Childhood eczema was associated only with maternal asthma during pregnancy and not with pre-pregnancy obesity, smoking during pregnancy, or antibiotic/antidepressant medication use during pregnancy. These findings are consistent with a Swedish study that concluded that maternal BMI was associated with an increased risk of asthma, but not with eczema or sensitisation in offspring [13].

This study found that children’s risk of having ever been diagnosed with asthma was positively associated with maternal asthma during pregnancy. This association was consistent and increased up to the age of 12–13 years. Among the children whose mothers had been experiencing asthma during pregnancy, the odds of having been diagnosed with asthma and the odds of having ongoing asthma were higher than the odds of having wheezing or eczema and consistently increased until the age of 10–11 years. A Danish study from a cohort of 675,379 singleton births (1996–2006) showed that children of mothers who had severe asthma during pregnancy had a higher prevalence of asthma (OR: 1.37; 95% CI: 1.17–1.61) compared with children of mothers with mild or no asthma during pregnancy [40]. Thus, this study reiterates previous findings of an association of maternal asthma during pregnancy with the risk of the offspring having asthma, but it also contributes further insight into the trend of asthma morbidity risks across age groups (birth to 15 years of age) among Australian children.

Children of mothers who were overweight or obese just before their pregnancy were highly likely to have at some point, been diagnosed with asthma. However, this likelihood diminished when they reached age 12 years. These findings corroborate earlier studies, one of which showed that United States children of obese mothers were 1.63 times more likely to have asthma than those of mothers with a healthy weight [23]. Though studies have revealed that gestational age and birth weight influence the risk of having asthma in early childhood [26], our study did not show any association with these confounding variables. These findings may imply a relationship between childhood obesity and asthma exposure among children but further research is needed [26, 43].

The adverse effects of smoking during pregnancy on childhood asthma are already evident in the existing literature [44]. Children may suffer from asthma morbidities due to exposure to environmental tobacco smoke or parental prenatal/postnatal smoking [42]. Although the rate of maternal cigarette smoking during pregnancy has decreased in the last decade in Australia [4], the evidence of the influence of smoking during pregnancy on childhood asthma persists [45]. However, few studies have investigated the number of cigarettes smoked during pregnancy and its effects on respiratory and allergic morbidities in their children [27]. This study investigated the influence of maternal smoking during pregnancy according to the category of number of cigarettes smoked. The prevalence of wheezing was higher among the children of mothers who smoked 11 or more cigarettes daily and was lower among the children of mothers who smoked fewer than ten cigarettes daily than the children of mothers who did not smoke during their pregnancy. The risk of asthma was only higher among the children of mothers who had 11 or more cigarettes daily during the first trimester of the pregnancy. Similarly, a Finish study (children born in 1987) found that mothers’ smoking, < 10 cigarettes per day or 11+ cigarettes per day during pregnancy, increased the probability of their offspring’s asthma [27].

Research on the association between maternal antibiotic or antidepressant use during pregnancy and respiratory and allergic morbidities in offspring is rare. Two studies found that maternal use of antibiotics in the third trimester of pregnancy, slightly increased the risk of their preschool-aged children having asthma [15, 33]. A Danish study of a birth cohort by Stockhome et al. [28] also found a causal effect but did not identify any trimester-specific effects of antibiotic use. In our study, except for wheezing among children of 2–5 years of age, no significant associations were observed between maternal medication use (antibiotics or antidepressants) during pregnancy and increased the risk of their offspring’s respiratory or allergic morbidities. Further, our findings corroborate with previous research on the use of modern antidepressants during pregnancy [16] which concluded that antidepressant use during pregnancy generally does not increase asthma risk. However, a study by Liu et al. of the Danish children, revealed that the use of an earlier variant of antidepressants during pregnancy [16] was associated with an increased risk of asthma among their offspring.

Our results indicate that maternal asthma during pregnancy increases the risk of children experiencing eczema, although in our sample, the prevalence of eczema was intermittent across the years until their adolescence. Significant risk of exposure was evident when the children were infants, aged 4–7 years, and again during adolescence at 10–13 years of age. However, these associations were not as strong as the children’s risk of encountering wheezing or asthma. A key finding of this study is that among the maternal health conditions during pregnancy, except for maternal asthma, none were associated with the risk of their children having eczema.

The age at onset of wheezing, asthma or eczema shows a pattern in our study with increasing prevalence in early childhood and decreasing prevalence during adolescents. Along with the maternal health risk factors revealed by our study, several other environmental risk factors showing association with these diseases revealed by other studies might be useful to explain the prevalence pattern [46, 47]. For example, a study demonstrates that early life sensitization to indoor allergens or mould is a predictor of asthma development [46]. Further, another study shows evidence that the indoor school environment is a significant reservoir of allergens, moulds, pollutants, and endotoxin and that there is an association between school exposure and pediatric asthma morbidity [47]. Previous studies also show that early childhood asthma is more common in children who are exposed to soot, exhaust, household tobacco smoking by household members, or oil smoke [48, 49]. Other studies have concluded that nylon clothing, unfamiliar pets, dust, and sweat are responsible for childhood eczema [50, 51]. All of these studies revealing the risk factors of influencing asthma or eczema are among the children in their early childhood, which are in line with the results of our study. Further, genetic influence on the age at onset of asthma may also explain the prevalence pattern or the association of risk factors of asthma. A study on Danish twins by Thomsen et al. in 2010 reveals that the risk of asthma in the co-twin decreases with increasing age at onset of asthma in the index twin [52]. Another biometric analysis study by Skadhauge et al. emphasized a major influence of genetic factors in the aetiology of asthma. However, a substantial part of the variation in liability to asthma is due to the impact of environmental factors specific to the individual. The study found no evidence for a substantial impact of genetic dominance or the shared environment [53].

Our study findings support gender dimorphism for both prevalence and risk factor associations of the respiratory and allergic diseases. As evidence of the gender differences, this study found that for both asthma and eczema boys had increased prevalence compared to girls in their early childhood, while it was reversed during their adolescence. Further, regarding the odds of having these diseases our study found that children whose mothers had asthma during pregnancy, boys had higher odds of having wheezing or asthma than girls until age 10–11 years. However, in the adolescent age group (12–15 years), the girls had higher odds of being ill with ongoing asthma compared to the boys. These study finding are supported by previous two studies conducted on asthma and puberty [54]. Findings of other studies also support a gender dimorphism in the obesity–asthma phenotype; they have found that the odds of asthma impairment related to obesity is highest among women aged 12 to 44 years of age [55, 56]. The gender differences may have been potentially linked to fluctuations of hormones during puberty and menstruation [54], physical activity levels, and eating habits [35]. These variations may also occur due to the differences in gender-specific responses to immunological, environmental, or occupational exposures [57–60].

A main strength of our study is its use of a large, nationally representative, ethnically inclusive, population-based birth cohort generalisable to all Australian children born in 2004. A range of maternal health indicators, including maternal asthma, pre-pregnancy obesity, gestational age, antibiotic use, and antidepressant use, were considered key explanatory variables. Adjustments were made for important confounders such as the children’s birth weight, breastfeeding status, housing environment and other socioeconomic covariates.

There are some limitations. First, the pre-pregnancy BMI was calculated using self-reported data on height and weight. Second, we did not have information about the severity of maternal asthma, genetic factors, or environmental exposures such as maternal mental health during pregnancy, infections during pregnancy, and maternal or child exposure to air pollutants. Thus, though we accounted for as many potential confounders as possible, our analyses were limited by the measurements available in the LSAC data. Future research could include other such factors that may exacerbate or mitigate the effects of maternal asthma, smoking, and other health status during pregnancy on their children’s respiratory or allergic outcomes.

## Conclusion

This longitudinal study revealed the prevalence trends of childhood wheezing, asthma, and eczema at 0–15 year of age and found increasing trend in early childhood and decreasing trend from 6 to 7 years to until their age of 14–15 years. Wheezing decreased to a greater extent than asthma and eczema. There were gender differences in the prevalence of these respiratory and allergic morbidities over time. This study also found that maternal asthma, obesity, and smoking during pregnancy were significantly associated with an increased risk of offspring’s wheezing or asthma. Only maternal asthma during pregnancy was significantly associated with the risk of eczema of their offspring. There were age and sex specific differences in the associations of maternal health or health risk factors with disease outcomes: a shift in the extent of the associations started at 6–7 years of age; where, at this age point, higher odds of both asthma and eczema were observed in female children. These findings have important public health implications for Australia. Our findings suggest that careful medical and obstetric monitoring, improved specific age-sex wise risk factor prevention where wheezing, asthma and eczema effect children and health promotion for pregnant women and children by the policy makers are highly warranted and may help protect child health.

## Data Availability

The data used for the study were collected from the Longitudinal Study of Australian Children Dataverse of National Centre for Longitudinal Data. Those interested in accessing this data should contact the Longitudinal Study of Australian Children Dataverseof National Centre for Longitudinal Data, Australia. There are some restrictions on the use of this data and the data application’s approval is subject to a signed confidentiality deed.

## Additional fles

**Additional file 1.** Appendix A; Description of Data: Details of the study participants and loss to follow-up analysis. **Additional file 2.** Appendix B; Description of Data: Longitudinal prevalence of wheezing, asthma, and eczema (tabular data of Fig. 1). **Additional file 3.** Appendix C; Description of Data: Sex segregated analysis tables on the risk of experiencing wheezing, asthma or eczema among children.

## Abbreviations

CI: Confidence Interval
GINA: Global Initiative for Asthma
LSAC: Longitudinal Study of Australian Children
OR: Odds Ratio
WHO: World Health Organization
