# Supplementary material for: Are wheezing, asthma and eczema in children associated with mother’s health during pregnancy? Evidence from an Australian birth cohort
Source: Arch Public Health. 2021 Nov 9;79:193. doi: 10.1186/s13690-021-00718-w (PMC8577022; doi:10.1186/s13690-021-00718-w)
Supplement: Supplementary file 1 — Additional file 1. Appendix A; Description of Data: Details of the study participants and loss to follow-up analysis. [file 13690_2021_718_MOESM1_ESM.docx]

**Appendix A: Details of the study participants**

**No. of non-mother Parents excluded***

**This Study Participants**

**Wave, Year**

**LSAC Participants**

Notes:

* Non-mother parents are those who responded as parent 1 in LSAC and had relationship to the study child as father, grandparent, adopted parent, step-parent, foster parent, aunt/uncle, sibling or unrelated adults in the respective waves.

** All attritions are calculated from the first wave to the particular wave.

Ϯ Loss to follow-up has been calculated from eligible sample of the first wave (baseline wave) to the particular wave.

Loss to follow-up: n = 2017

Loss to follow-up: n = 1880

Loss to follow-up: n = 1403

Loss to follow-up: n = 1055

Loss to follow-up: n = 889

Loss to follow-up: n = 713

**Ϯ** Loss to follow-up: n = 492

Mothers, n= 4977

Children, n = 4977

Mothers, n= 4485

Children, n = 4485

Mothers, n= 4264

Children, n = 4264

Mothers, n= 4088

Children, n = 4088

Mothers, n= 3922

Children, n = 3922

Mothers, n= 3574

Children, n = 3574

Mothers, n= 3097

Children, n = 3097

Mother, n= 2960

Children, n = 2960

n= 167

n= 190

n= 284

n= 130

n= 121

n= 122

n= 154

n= 163

Parents, n= 3127

Children, n = 3127

Parents, n= 3381

Children, n = 3381

Parents, n= 3764

Children, n = 3764

Parents, n= 4085

Children, n = 4085

Parents, n= 4242

Children, n = 4242

Parents, n= 4386

Children, n = 4386

Parents, n= 4606

Children, n = 4606

Parents, n= 5107

Children, n = 5107

Attrition: 38.8%, n = 1980

Attrition: 33.8%, n = 1726

Attrition: 26.3%, n = 1343

Attrition: 20.0%, n = 1022

Attrition: 16.9%, n = 865

Attrition: 14.1%, n = 721

**Attrition: 9.8%, n = 501

**Figure A1: Participant diagram**

Wave 1, 2004

Wave 7, 2016

Wave 8, 2018

Wave 6, 2014

Wave 5, 2012

Wave 4, 2010

Wave 3, 2008

Wave 2, 2006

Table A1: Loss of follow-up analysis of the prevalence of wheezing, ever diagnosed with asthma, ongoing asthma and eczema

| **Timeline** | **Wheezing** | **Ever had Asthma** | **Currently had asthma** | **Eczema** |
| --- | --- | --- | --- | --- |
|  | n (%) | n (%) | n (%) | n (%) |
| Baseline at Age 0-1 year (Wave-1) | 820 (16.47) |  |  | 729 (14.65) |
| Baseline at Age 2-3 year (for asthma, Wave-2) |  | 647 (14.43) | 523 (11.65) |  |
| Loss to follow-up at Age 2-3 year (Wave-2) |  |  |  |  |
| *With respect to baseline wave* | 84 (17.06) | - | - | 71 (14.51) |
| Loss to follow-up at Age 4-5 year (Wave-3) |  |  |  |  |
| *With respect to baseline wave* | 140 (19.62) | 65 (18.21) | 54 (15.00) | 96 (13.54) |
| *With respect to Wave-2* | 98 (27.79) | 67 (18.95) | 55 (15.76) | 43 (12.28) |
| Loss to follow-up at Age 6-7 year (Wave-4) |  |  |  |  |
| *With respect to baseline wave* | 189 (21.24) | 109 (19.69) | 87 (15.67) | 117 (13.11) |
| *With respect to Wave-3* | 65 (22.24) | 76 (26.17) | 47 (16.32) | 39 (13.42) |
| Loss to follow-up at Age 8-9 year (Wave-5) |  |  |  |  |
| *With respect to baseline wave* | 209 (19.88) | 114 (16.3) | 94 (13.36) | 147 (13.97) |
| *With respect to Wave-4* | 58 (19.05) | 86 (28.29) | 38 (12.50) | 45 (14.80) |
| Loss to follow-up at Age 10-11 year (Wave-6) |  |  |  |  |
| *With respect to baseline wave* | 271 (19.33) | 171 (16.69) | 145 (14.13) | 183 (13.02) |
| *With respect to Wave-5* | 56 (12.72) | 107 (24.48) | 59 (13.47) | 48 (10.93) |
| Loss to follow-up at Age 12-13 year (Wave-7) |  |  |  |  |
| *With respect to baseline wave* | 357 (19.00) | 224 (15.97) | 185 (13.21) | 253 (13.46) |
| *With respect to Wave-6* | 84 (14.40) | 193 (33.01) | 89 (15.26) | 89 (15.10) |
| Loss to follow-up at Age 14-15 year (Wave-8) |  |  |  |  |
| *With respect to baseline wave* | 374 (18.53) | 256 (15.96) | 215 (13.36) | 272 (13.50) |
| *With respect to Wave-7* | 23 (6.62) | 114 (33.17) | 38 (11.08) | 55 (15.85) |

Table A2: Loss of follow-up analysis of the baseline characteristics of the explanatory variables of the sampled children

|  | **Baseline at Age**  **0-1 year** | **Loss to follow-up at Age 2-3 year** | **Loss to follow-up at Age 4-5 year** | **Loss to follow-up at Age 6-7 year** | **Loss to follow-up at Age 8-9 year** | **Loss to follow-up at Age 10-11 year** | **Loss to follow-up at Age 12-13 year** | **Loss to follow-up at Age 14-15 year** |
| --- | --- | --- | --- | --- | --- | --- | --- | --- |
| **VARIABLES** | **n (%)** | **n (%)** | **n (%)** | **n (%)** | **n (%)** | **n (%)** | **n (%)** | **n (%)** |
| **Number of children/mothers 🡪** | 4977 | 492 | 713 | 889 | 1055 | 1403 | 1880 | 2017 |
| **Explanatory Variables** |  |  |  |  |  |  |  |  |
| **Mother had asthma during pregnancy** |  |  |  |  |  |  |  |  |
| *No* | 4619 (92.81) | 452 (91.89) | 662 (92.73) | 822 (92.42) | 990 (93.76) | 1311 (93.41) | 1759 (93.58) | 1892 (93.78) |
| *Yes* | 358 (7.19) | 40 (8.11) | 51 (7.29) | 67 (7.58) | 65 (6.24) | 92 (6.59) | 121 (6.42) | 125 (6.22) |
| **Pre-pregnancy Obesity of Mother** |  |  |  |  |  |  |  |  |
| *Underweight* | 469 (9.42) | 40 (8.02) | 64 (8.98) | 81 (9.16) | 97 (9.19) | 126 (8.96) | 183 (9.71) | 194 (9.61) |
| *Healthy weight* | 1786 (35.89) | 108 (22.02) | 169 (23.76) | 218 (24.52) | 270 (25.54) | 384 (27.38) | 521 (27.71) | 581 (28.78) |
| *Overweight* | 1007 (20.23) | 64 (12.96) | 83 (11.59) | 128 (14.41) | 165 (15.58) | 218 (15.54) | 319 (16.96) | 353 (17.52) |
| *Obesity* | 714 (14.35) | 43 (8.83) | 80 (11.19) | 99 (11.14) | 118 (11.27) | 174 (12.44) | 242 (12.9) | 264 (13.08) |
| *Not measured* | 1001 (20.11) | 237 (48.16) | 317 (44.48) | 362 (40.76) | 406 (38.42) | 501 (35.69) | 615 (32.72) | 625 (31.01) |
| **Gestational age at birth** |  |  |  |  |  |  | . |  |
| *On time (37–41 weeks)* | 4407 (88.55) | 421 (85.48) | 612 (85.86) | 772 (86.88) | 917 (86.89) | 1212 (86.39) | 1632 (86.82) | 1765 (87.5) |
| *Early (36 weeks or less)* | 338 (6.80) | 45 (9.24) | 58 (8.08) | 71 (8.03) | 88 (8.33) | 117 (8.33) | 146 (7.73) | 159 (7.89) |
| *Late (42 weeks or more)* | 232 (4.65) | 26 (5.29) | 43 (6.06) | 45 (5.09) | 50 (4.77) | 74 (5.28) | 102 (5.44) | 93 (4.61) |
| **Mother ever smoked during pregnancy** | |  |  |  |  |  |  |  |
| *No* | 4224 (84.88) | 410 (83.33) | 586 (82.14) | 733 (82.49) | 856 (81.14) | 1140 (81.22) | 1547 (82.27) | 1644 (81.50) |
| *Yes* | 753 (15.12) | 82 (16.67) | 127 (17.86) | 156 (17.51) | 199 (18.86) | 263 (18.78) | 333 (17.73) | 373 (18.50) |
| **Mother’s smoking during 1st trimester** | |  |  |  |  |  |  |  |
| *None* | 4347 (87.35) | 426 (86.57) | 604 (84.66) | 753 (84.68) | 883 (83.72) | 1178 (83.98) | 1602 (85.22) | 1707 (84.65) |
| *<=10 cigarettes daily* | 410 (8.23) | 45 (9.09) | 72 (10.09) | 90 (10.10) | 109 (10.32) | 139 (9.90) | 179 (9.5) | 197 (9.78) |
| *11+ cigarettes daily* | 220 (4.42) | 21 (4.33) | 37 (5.24) | 46 (5.22) | 63 (5.96) | 86 (6.13) | 99 (5.28) | 112 (5.57) |
| **Antidepressant medication during pregnancy** |  |  |  |  |  |  |  |  |
| *No* | 4870 (97.86) | 484 (98.30) | 700 (98.16) | 879 (98.83) | 1031 (97.67) | 1371 (97.71) | 1838 (97.76) | 1958 (97.08) |
| *Yes* | 107 (2.14) | 8 (1.70) | 13 (1.84) | 10 (1.17) | 24 (2.31) | 32 (2.29) | 42 (2.24) | 59 (2.92) |
| **Antibiotic medication during pregnancy** |  |  |  |  |  |  |  |  |
| *No* | 4452 (89.46) | 440 (89.53) | 636 (89.19) | 789 (88.78) | 950 (89.98) | 1253 (89.32) | 1684 (89.57) | 1810 (89.76) |
| *Yes* | 525 (10.54) | 52 (10.47) | 77 (10.81) | 100 (11.22) | 105 (10.02) | 150 (10.68) | 196 (10.43) | 207 (10.24) |
| **CONTROL VARIABLES** |  |  |  |  |  |  |  |  |
| **Child Health Issues** |  |  |  |  |  |  |  |  |
| **Birth weight** |  |  |  |  |  |  |  |  |
| *Normal (2,500–3,999)* | 4071 (81.79) | 399 (81.05) | 587 (82.35) | 733 (82.49) | 881 (83.46) | 1175 (83.71) | 1559 (82.92) | 1667 (82.67) |
| *Low (<2,500)* | 279 (5.61) | 50 (10.25) | 50 (7.03) | 62 (6.96) | 77 (7.29) | 89 (6.36) | 115 (6.1) | 126 (6.25) |
| High (>=4,000) | 627 (12.59) | 43 (8.7) | 76 (10.62) | 94 (10.55) | 97 (9.26) | 139 (9.93) | 206 (10.97) | 224 (11.09) |
| **Immunisation status of children** |  |  |  |  |  |  |  |  |
| *Up to date* | 4516 (90.74) | 415 (84.31) | 615 (86.20) | 790 (88.87) | 943 (89.38) | 1244 (88.68) | 1670 (88.82) | 1799 (89.22) |
| *Not up to date* | 461 (9.26) | 77 (15.69) | 98 (13.80) | 99 (11.13) | 112 (10.62) | 159 (11.32) | 210 (11.18) | 218 (10.78) |
| **Breastfed children up to 6 months** |  |  |  |  |  |  |  |  |
| *Yes* | 2328 (46.78) | 185 (37.61) | 260 (36.42) | 322 (36.24) | 395 (37.45) | 526 (37.49) | 722 (38.41) | 779 (38.60) |
| *No* | 2649 (53.22) | 307 (62.39) | 453 (63.58) | 567 (63.76) | 660 (62.55) | 877 (62.51) | 1158 (61.59) | 1238 (61.40) |
| **Mother’s sleep quality in the year of childbirth** | |  |  |  |  |  |  |  |
| *Very good/Fairly good* | 3478 (69.89) | 342 (69.56) | 500 (70.08) | 619 (69.61) | 739 (69.98) | 987 (70.34) | 1323 (70.36) | 1417 (70.24) |
| *Fairly bad* | 1118 (22.46) | 113 (22.96) | 153 (21.52) | 195 (21.89) | 220 (20.87) | 301 (21.49) | 404 (21.5) | 437 (21.67) |
| *Very bad* | 381 (7.66) | 37 (7.48) | 60 (8.40) | 75 (8.5) | 96 (9.15) | 115 (8.17) | 153 (8.14) | 163 (8.09) |
