# Supplementary material for: Are wheezing, asthma and eczema in children associated with mother’s health during pregnancy? Evidence from an Australian birth cohort
Source: Arch Public Health. 2021 Nov 9;79:193. doi: 10.1186/s13690-021-00718-w (PMC8577022; doi:10.1186/s13690-021-00718-w)
Supplement: Supplementary file 2 — Additional file 2. Appendix B; Description of Data: Longitudinal prevalence of wheezing, asthma, and eczema (tabular data of Fig. 1). [file 13690_2021_718_MOESM2_ESM.docx]

**Appendix B: Longitudinal prevalence of wheezing, asthma, and eczema (tabular data of Figure 2)**

Table B-1: Prevalence of wheezing by age group and sex

| Age Group | All | Male | Female |
| --- | --- | --- | --- |
| 0-1 | 16.5% | 18.6% | 14.2% |
| 2-3 | 26.0% | 28.0% | 23.8% |
| 4-5 | 20.0% | 21.3% | 18.7% |
| 6-7 | 18.6% | 19.8% | 17.4% |
| 8-9 | 12.8% | 14.7% | 10.8% |
| 10-11 | 11.3% | 12.0% | 10.6% |
| 12-13 | 9.3% | 10.4% | 8.0% |
| 14-15 | 7.3% | 7.67% | 7.03% |

Table B-2: Prevalence of having ever diagnosed with asthma by age group and sex

| Age Group | All | Male | Female |
| --- | --- | --- | --- |
| 2-3 | 14.4% | 17.3% | 11.5% |
| 4-5 | 21.6% | 24.5% | 18.7% |
| 6-7 | 26.3% | 28.9% | 22.9% |
| 8-9 | 27.0% | 29.6% | 23.8% |
| 10-11 | 30.5% | 33.0% | 26.8% |
| 12-13 | 31.5% | 33.5% | 28.1% |
| 14-15 | 32.3% | 33.6% | 29.8% |

Table B-3 Prevalence of ongoing asthma by age group and sex

| Age Group | All | Male | Female |
| --- | --- | --- | --- |
| 2-3 | 11.7% | 13.6% | 9.7% |
| 4-5 | 13.4% | 15.9% | 12.7% |
| 6-7 | 15.3% | 16.8% | 13.8% |
| 8-9 | 15.3% | 16.8% | 13.7% |
| 10-11 | 15.4% | 17.0% | 13.7% |
| 12-13 | 14.8% | 15.3% | 14.3% |
| 14-15 | 13.6% | 13.46% | 13.7% |

Table B-4 Prevalence of eczema by age group and sex

| Age Group | All | Male | Female |
| --- | --- | --- | --- |
| 0-1 | 14.7% | 15.3% | 14.0% |
| 2-3 | 17.8% | 18.5% | 17.0% |
| 4-5 | 14.7% | 14.8% | 14.6% |
| 6-7 | 13.2% | 12.1% | 14.3% |
| 8-9 | 12.7% | 10.9% | 14.6% |
| 10-11 | 11.4% | 9.8% | 13.2% |
| 12-13 | 10.0% | 8.1% | 12.0% |
| 14-15 | 9.5% | 7.0% | 12.1% |
