# Supplementary material for: Are wheezing, asthma and eczema in children associated with mother’s health during pregnancy? Evidence from an Australian birth cohort
Source: Arch Public Health. 2021 Nov 9;79:193. doi: 10.1186/s13690-021-00718-w (PMC8577022; doi:10.1186/s13690-021-00718-w)
Supplement: Supplementary file 3 — Additional file 3. Appendix C; Description of Data: Sex segregated analysis tables on the risk of experiencing wheezing, asthma or eczema among children. [file 13690_2021_718_MOESM3_ESM.docx]

Appendix C: Sex segregated analysis tables on the risk of experiencing wheezing, asthma or eczema among children

**Table C1**: The risk of experiencing wheezing among male children based on the incidence of maternal asthma, other morbidities, and maternal health behaviours during pregnancy

| **Maternal health, risk factors, and medications during pregnancy** | **Age 0-1** | **Age 2-3** | **Age 4-5** | **Age 6-7** | **Age 8-9** | **Age 10-11** | **Age 12-13** | **Age 14-15** |
| --- | --- | --- | --- | --- | --- | --- | --- | --- |
|  | OR (95% CI) | OR (95% CI) | OR (95% CI) | OR (95% CI) | OR (95% CI) | OR (95% CI) | OR (95% CI) | OR (95% CI) |
|  | N = 4977 | N = 4485 | N = 4264 | N = 4088 | N = 3922 | N = 3574 | N = 3097 | N = 2960 |
| **Had asthma** |  |  |  |  |  |  |  |  |
| *No* |  |  |  |  |  |  |  |  |
| *Yes* | 1.50 (1.03-2.18)* | 1.93 (1.35-2.76)** | 1.77 (1.20-2.63)^*^ | 2.07 (1.35-3.18)* | 1.97 (1.23-3.18)** | 3.28 (2.01-5.34)** | 1.56 (0 .78 -3.11) | 1.13 (0.49-2.61) |
| **Gestational age at birth** |  |  |  |  |  |  |  |  |
| *On time (37-41 weeks, ref.)* |  |  |  |  |  |  |  |  |
| *Early (36 weeks or less)* | 1.25 (0.78-1.99) | 1.54 (0.97-2.45)^Ϯ^ | 1.50 (0.90-2.48) | 1.96 (1.19-3.23) ^Ϯ^ | 1.26 (0.67-2.36) | 1.03 (0.41-2.63) | 0.35 (0.11-1.09) ^Ϯ^ | 1.15 (0.47-2.85) |
| *Late (42 weeks or more)* | 0.77 (0.43-1.36) | .92 (0.56-1.51) | 1.34 (0.78-2.31) | 1.33 (0.77-2.27) | 1.59 (0.87-2.91) | 1.31 (0.59-2.89) | 1.63 (0.76-3.52) | 1.46 (0.58-3.67) |
| **Pre-pregnancy obesity** |  |  |  |  |  |  |  |  |
| *Healthy weight* |  |  |  |  |  |  |  |  |
| *Underweight* | 0.38 (0.23-0.63)** | 0.76 (0.50-1.16) | 0.60 (0.36-.99) | 0.72 (0.45-1.15) | 0.69 (0.39-1.23) | 0.64 (0.31-1.28) | 1.32 (0.66-2.62) | 0.55 (0.23-1.36) |
| *Overweight* | 1.06 (0.77-1.45) | 1.66 (1.26-2.20)** | 0.98 (0.72-1.36) | 1 (0.71-1.41) | 1.04 (0.71-1.52) | 0.93 (0.56-1.53) | 2.08 (1.21-3.58) ^Ϯ^ | 1.29 (0.70-2.39) |
| *Obesity* | 0.97 (0.68-1.38) | 1.56 (1.14-2.15)^Ϯ^ | 1.23 (0.86-1.75) | 1.03 (0.70-1.51) | 0.99 (0.64-1.53) | 1.24 (0.77-2.01) | 1 (0.53-1.91) | 0.97 (0.50-1.90) |
| *Not known* | 1.66 (1.22-2.24)* | 1.52 (1.11-2.07)^Ϯ^ | 1.41 (1-2)^Ϯ^ | 1.46 (1.01-2.10)* | 1.19 (0.80-1.77) | 1.23 (0.73-2.05) | 1.47 (0.81-2.67)* | 1.81 (0.95-3.43)^Ϯ^ |
| **Smoking during 1^st^ trimester** |  |  |  |  |  |  |  |  |
| *None (ref.)* |  |  |  |  |  |  |  |  |
| *<=10 cigarettes daily* | 1.75 (1.19-2.57)* | 1.50 (1.04-2.18)* | 1.40 (0.92-2.14) | 1.31 (0.83-2.08) | 1.24 (0.75-2.06) | 0.96 (0 .47 -1.95) | 1.74 (0. 89 -3.41) | 1.45 (0.61-3.45) |
| *11+ cigarettes daily* | 1.37 (0.83-2.25) | 1.28 (0.76-2.16) | 1.99(1.1-3.60)* | 2.71 (1.51-4.85)* | 1.33 (0.66-2.69) | 1.10 (0.48-2.54) | 0.73 (0.23-2.29) | 1.72 (0.58-5.07)* |
| **Antibiotic medication** |  |  |  |  |  |  |  |  |
| *No* |  |  |  |  |  |  |  |  |
| *Yes* | 1.39 (0.98-1.96) Ϯ | 1.33 (0.95-1.87) ^Ϯ^ | 1.25 (0.83-1.86) | 1.14 (0.75-1.73) | 1.32 (0.85-2.07) | 1.19 (0.65-2.19) | 1.37 (0.68-2.75) | 0.68 (0.27-1.68) |
| **Anti-depressant medication** |  |  |  |  |  |  |  |  |
| *No* |  |  |  |  |  |  |  |  |
| *Yes* | 1.70 (0.84-3.44) | 2.08 (1.10-3.94)* | 0.99 (0.47-2.07) | 1.07 (0.50-2.32) | 0.38 (0.12-1.18) ^Ϯ^ | 1.80 (0.66-4.86) | 2.09 (0.65-6.73) | - |

Notes: Ϯ: p<0.05 & >0.01; *: p<0.01 & >0.001; **: p<0.001; the regression models were adjusted for several covariates outlined in ‘Control variables’ sub-section of ‘Methods’ section.

**Table C2**: The risk of experiencing wheezing among female children based on the incidence of maternal asthma, other morbidities, and maternal health behaviours during pregnancy

| **Maternal health, risk factors, and medications during pregnancy** | **Age 0-1** | **Age 2-3** | **Age 4-5** | **Age 6-7** | **Age 8-9** | **Age 10-11** | **Age 12-13** | **Age 14-15** |
| --- | --- | --- | --- | --- | --- | --- | --- | --- |
|  | OR (95% CI) | OR (95% CI) | OR (95% CI) | OR (95% CI) | OR (95% CI) | OR (95% CI) | OR (95% CI) | OR (95% CI) |
|  | N = 4977 | N = 4485 | N = 4264 | N = 4088 | N = 3922 | N = 3574 | N = 3097 | N = 2960 |
| **Had asthma** |  |  |  |  |  |  |  |  |
| *No* |  |  |  |  |  |  |  |  |
| *Yes* | 1.46 (0.95-2.24) ^Ϯ^ | 1.73 (1.14-2.65)* | 1.15 (0.70-1.91) | 1.59 (0.96-2.66) ^Ϯ^ | 1.80 (1.03-3.15)* | 1.72 (0.92-3.21) ^Ϯ^ | 1.67 (0.81-3.46) | 1.48 (0.69-3.15) |
| **Gestational age at birth** |  |  |  |  |  |  |  |  |
| *On time (37-41 weeks, ref.)* |  |  |  |  |  |  |  |  |
| *Early (36 weeks or less)* | 1.53 (0.92-2.57) | 1.55 (0.90-2.67) | 1.23 (0.67-2.26) | 1.04 (0.47-2.28) | 0.82 (0.38-1.75) | 0.50 (0.18-1.37) | 0.70 (0.15-2.94) | 1.04 (0.24-4.41) |
| *Late (42 weeks or more)* | 0.46 (0.23-0.93)* | 1.06 (0.62-1.83) | 1.89 (1.03-3.50)* | 0.84 (0.38-1.77) | 0.41 (0.16-1.06) ^Ϯ^ | 1.10 (0.48-2.56) | 0.84 (0.23-3.15) | 0.55 (0.15-2.03) |
| **Pre-pregnancy obesity** |  |  |  |  |  |  |  |  |
| *Healthy weight* |  |  |  |  |  |  |  |  |
| *Underweight* | 1.22 (0.76-1.97) | 1.05 (0.70-1.58) | 1.31 (0.81-2.11) | 0.84 (0.49-1.45) | 1.05 (0.56-1.96) | 1.42 (0.72-2.82) | 0.88 (0.36-2.13) | 0.63 (0.19-2.07) |
| *Overweight* | 1.40 (0.98-1.98) ^Ϯ^ | 1.18 (0.85-1.60) | 1.30 (0.93-1.84) | 1.06 (0.73-1.54) | 1.58 (1.01-2.49)* | 1.47 (0.88-2.46) | 0.90 (0.47-1.73) | 1.32 (0.70-2.49) |
| *Obesity* | 1.55 (1.03-2.33)* | 1.24 (0.88-1.74) | 1.47 (1.01-2.17)^Ϯ^ | 1.45 (0.97-2.16) ^Ϯ^ | 1.70 (1.03-2.79)* | 2.20 (1.29-3.73)^**^ | 2.14 (1.09-4.20)* | 1.73 (0.87-3.43) |
| *Not known* | 1.37 (0.96-1.96) ^Ϯ^ | 1.58 (1.14-2.17)** | 1.28 (0.86-1.90) | 1.30 (0.87-1.95) | 1.22 (0.76-1.98) | 1.78 (1.03-3.06)* | 2.14 (1.13-4.06)* | 1.93 (0.98-3.80)* |
| **Smoking during 1^st^ trimester** |  |  |  |  |  |  |  |  |
| *None (ref.)* |  |  |  |  |  |  |  |  |
| *<=10 cigarettes daily* | 1.45 (0.92-2.27) | 1.58 (1.05-2.38)* | 1.37 (0.86-2.18) | 1.95 (1.20-3.15)** | 1.47 (0.78-2.75) | 1.09 (0.54-2.18) | 0.69 (0.25-1.92) | 0.89 (0.36-2.35) |
| *11+ cigarettes daily* | 1.33 (0.75-2.36) | 2.31 (1.38-3.89)** | 1.68 (0.96-2.94) ^Ϯ^ | 1.35 (0.71-2.55) | 1.42 (0.66-3.06) | 2.13 (1-4.55)* | 1.55 (0.53-4.49) | 3.5 (1.43-8.79)** |
| **Antibiotic medication** |  |  |  |  |  |  |  |  |
| *No* |  |  |  |  |  |  |  |  |
| *Yes* | 1.16 (0.80-1.67) | 1.47 (1.06-2.06)* | 1.74 (0.79-1.78) | 1.18 (0.87-1.55) | 0.88 (0.55-1.47) | 1.40 (0.86-2.27) | 0.90 (0.43-1.86) | 1.43 (0.74-2.76) |
| **Anti-depressant medication** |  |  |  |  |  |  |  |  |
| *No* |  |  |  |  |  |  |  |  |
| *Yes* | 0.87 (0.36-2.09) | 1.65 (0.77-3.52) | 1.19 (0.53-3.11) | 1.29 (0.65-2.11) | 1.18 (0.42-3.36) | 1.39 (0.49-4) | 0.77 (0.16-3.64) | 2.36 (0.75-7.50) |

Notes: Ϯ: p<0.05 & >0.01; *: p<0.01 & >0.001; **: p<0.001; the regression models were adjusted for several covariates outlined in ‘Control variables’ sub-section of ‘Methods’ section.

**Table C3**: The risk of having ever been diagnosed with asthma among male children based on the incidence of maternal asthma, other morbidities, and health behaviours during pregnancy (cumulative effect over age)

| **Maternal health, Risk factors, and medications during pregnancy** | **Age 2-3** | **Age 4-5** | **Age 6-7** | **Age 8-9** | **Age 10-11** | **Age 12-13** | **Age 14-15** |
| --- | --- | --- | --- | --- | --- | --- | --- |
|  | OR (95% CI) | OR (95% CI) | OR (95% CI) | OR (95% CI) | OR (95% CI) | OR (95% CI) | OR (95% CI) |
|  | N = 4485 | N = 4264 | N = 4088 | N = 3922 | N = 3574 | N = 3097 | N = 2960 |
| **Had Asthma** |  |  |  |  |  |  |  |
| *No* |  |  |  |  |  |  |  |
| *Yes* | 2.76 (1.86-4.11)** | 2.75 (1.86-4.07)** | 2.69 (1.81-3.98)** | 2.87 (1.93-4.28)** | 3.04 (1.98-4.67)** | 2.66 (1.69-4.18)** | 2.35 (1.46-3.79)** |
| **Gestational age at birth** |  |  |  |  |  |  |  |
| *On time (37-41 weeks, ref.)* |  |  |  |  |  |  |  |
| *Early (36 weeks or less)* | 2.16 (1.25-3.72)** | 2.10 (1.30-3.40)** | 1.65 (1.02-2.65)* | 1.62 (0.98-2.66)* | 1.15 (0.68-1.95) | 0.83 (0.44-1.59) | 0.90 (0.47-1.72) |
| *Late (42 weeks or more)* | 1.07 (0.61-1.85) | 1.08 (0.62-1.88) | 0.79 (0.46-1.34) | 0.79 (0.46-1.34) | 0.83 (0.47-1.47) | 1.13 (0.61-2.07) | 1.07 (0.58-1.98) |
| **Pre-pregnancy obesity** |  |  |  |  |  |  |  |
| *Healthy weight* |  |  |  |  |  |  |  |
| *Underweight* | 1.06 (0.66-1.71) | 0.88 (0.57-1.36) | 0.99 (0.66-1.48) | 1.02 (0.69-1.5) | 1.00 (0.67-1.49) | 1.03 (0.66-1.61) | 1.10 (0.7-1.7) |
| *Overweight* | 1.3 (0.91-1.84) | 1.15 (0.84-1.58) | 1.36 (1.02-1.82) | 1.32 (0.99-1.77) | 1.19 (0.87-1.61) | 1.08 (0.77-1.51) | 1.20 (0.85-1.7) |
| *Obesity* | 1.65 (1.13-2.41)* | 1.45 (1.03-2.04)* | 1.34 (0.97-1.86)Ϯ | 1.38 (1.00-1.91)* | 1.23 (0.87-1.74) | 0.9 (0.62-1.3) | 1.00 (0.69-1.44) |
| *Not known* | 1.64 (1.14-2.36) | 1.54 (1.11-2.14) | 1.42 (1.02-1.96)* | 1.46 (1.05-2.02)* | 1.61 (1.13-2.28)** | 1.65 (1.12-2.43) | 1.44 (0.96-2.14)Ϯ |
| **Smoking during 1^st^ trimester** |  |  |  |  |  |  |  |
| *None (ref.)* |  |  |  |  |  |  |  |
| *<=10 cigarettes daily* | 1.02 (0.66-1.59) | 1.01 (0.67-1.53) | 1.16 (0.77-1.74) | 1.06 (0.70-1.61) | 1.25 (0.81-1.95) | 1.08 (0.66-1.75) | 1.03 (0.63-1.71) |
| *11+ cigarettes daily* | 1.59 (0.89-2.84) | 1.86 (1.07-3.22)* | 1.54 (0.89-2.68) | 1.26 (0.69-2.28) | 1.52 (0.82-2.81) | 1.4 (0.7-2.81) | 1.51 (0.77-2.97) |
| **Antibiotic medication** |  |  |  |  |  |  |  |
| *No* |  |  |  |  |  |  |  |
| *Yes* | 1.04 (0.69-1.58) | 1.03 (0.71-1.51) | 1.20 (0.81-1.76) | 1.38 (0.95-1.99)Ϯ | 1.28 (0.86-1.88) | 1.12 (0.73-1.73) | 1.00 (0.64-1.54) |
| **Anti-depressant medication** |  |  |  |  |  |  |  |
| *No* |  |  |  |  |  |  |  |
| *Yes* | 0.73 (0.28-1.92) | 1.09 (0.50-2.38) | 1.29 (0.64-2.6) | 1.00 (0.43-2.32) | 1.37 (0.58-3.23) | 1.18 (0.44-3.13) | 0.45 (0.11-1.77) |

Notes: Ϯ: p<0.05 & >0.01; *: p<0.01 & >0.001; **: p<0.001; the regression models were adjusted for several covariates outlined in ‘Control variables’ sub-section of ‘Methods’ section.

**Table C4**: The risk of having ever been diagnosed with asthma among female children based on the incidence of maternal asthma, other morbidities, and health behaviours during pregnancy (cumulative effect over age)

| **Maternal health, Risk factors, and medications during pregnancy** | **Age 2-3** | **Age 4-5** | **Age 6-7** | **Age 8-9** | **Age 10-11** | **Age 12-13** | **Age 14-15** |
| --- | --- | --- | --- | --- | --- | --- | --- |
|  | OR (95% CI) | OR (95% CI) | OR (95% CI) | OR (95% CI) | OR (95% CI) | OR (95% CI) | OR (95% CI) |
|  | N = 4485 | N = 4264 | N = 4088 | N = 3922 | N = 3574 | N = 3097 | N = 2960 |
| **Had Asthma** |  |  |  |  |  |  |  |
| *No* |  |  |  |  |  |  |  |
| *Yes* | 1.77 (1.1-2.86)* | 2.01 (1.31-3.10)** | 2.45 (1.57-3.81)** | 1.98 (1.30-3.01)** | 2.29 (1.46-3.59)** | 3.42 (2.06-5.69)** | 2.83 (1.73-4.62)** |
| **Gestational age at birth** |  |  |  |  |  |  |  |
| *On time (37-41 weeks, ref.)* |  |  |  |  |  |  |  |
| *Early (36 weeks or less)* | 0.60 (0.26-1.37) | 0.62 (0.32-1.20) | 0.5 (0.25-0.97)* | 0.93 (0.5-1.75) | 0.99 (0.50-1.99) | 0.76 (0.36-1.59) | 0.82 (0.41-1.65) |
| *Late (42 weeks or more)* | 0.34 (0.12-0.93)* | 0.90 (0.48-1.70) | 1.01 (0.53-1.89) | 0.88 (0.48-1.60) | 0.84 (0.43-1.64) | 1.05 (0.50-2.21) | 0.92 (0.46-1.81) |
| **Pre-pregnancy obesity** |  |  |  |  |  |  |  |
| *Healthy weight* |  |  |  |  |  |  |  |
| *Underweight* | 0.87 (0.50-1.52) | 0.80 (0.49-1.31) | 0.74 (0.46-1.2) | 0.68 (0.41-1.10) | 0.61 (0.36-1.02)Ϯ | 0.62 (0.36-1.05)Ϯ | 0.68 (0.39-1.18) |
| *Overweight* | 1.47 (0.99-2.17)* | 1.50 (1.08-2.10)* | 1.2 (0.87-1.65) | 1.25 (0.91-1.71) | 1.42 (1.02-1.96)* | 1.43 (1.01-2.02)* | 1.25 (0.88-1.78) |
| *Obesity* | 1.47 (0.93-2.31)Ϯ | 1.55 (1.07-2.24)* | 1.52 (1.05-2.2)* | 1.51 (1.06-2.14)* | 1.72 (1.19-2.48)** | 1.73 (1.16-2.59)** | 1.61 (1.06-2.43)* |
| *Not known* | 1.11 (0.72-1.71) | 1.11 (0.76-1.62) | 1.32 (0.91-1.9) | 1.25 (0.87-1.78) | 1.67 (1.14-2.44)** | 1.59 (1.05-2.41)* | 1.91 (1.27-2.87)** |
| **Smoking during 1^st^ trimester** |  |  |  |  |  |  |  |
| *None (ref.)* |  |  |  |  |  |  |  |
| *<=10 cigarettes daily* | 1.18 (0.68-2.04) | 0.80 (0.49-1.33) | 0.9 (0.54-1.49) | 0.87 (0.53-1.43) | 0.89 (0.52-1.54) | 0.92 (0.52-1.63) | 0.69 (0.35-1.35) |
| *11+ cigarettes daily* | 1.73 (0.95-3.17)Ϯ | 1.82 (1.03-3.20)* | 1.87 (1.05-3.31)* | 1.88 (1.05-3.35)* | 1.83 (0.97-3.45)Ϯ | 1.68 (0.85-3.31) | 2.00 (0.97-4.12)* |
| **Antibiotic medication** |  |  |  |  |  |  |  |
| *No* |  |  |  |  |  |  |  |
| *Yes* | 1.43 (0.96-2.15)Ϯ | 1.12 (0.77-1.62) | 1.18 (0.82-1.71) | 1.00 (0.70-1.43) | 1.10 (0.76-1.60) | 0.92 (0.60-1.41) | 1.09 (0.71-1.67) |
| **Anti-depressant medication** |  |  |  |  |  |  |  |
| *No* |  |  |  |  |  |  |  |
| *Yes* | 1.06 (0.42-2.65) | 1.57 (0.72-3.46) | 2.43 (1.13-5.24)* | 1.73 (0.79-3.80) | 1.65 (0.75-3.61) | 1.45 (0.56-3.75) | 0.68 (0.26-1.82) |

Notes: Ϯ: p<0.05 & >0.01; *: p<0.01 & >0.001; **: p<0.001; the regression models were adjusted for several covariates outlined in ‘Control variables’ sub-section of ‘Methods’ section.

**Table C5**: The risk of having ongoing asthma among male children based on the incidence of maternal asthma, other morbidities, and health behaviours during pregnancy

| **Maternal health, Risk factors, and medications during pregnancy** | **Age 2-3** | **Age 4-5** | **Age 6-7** | **Age 8-9** | **Age 10-11** | **Age 12-13** | **Age 14-15** |
| --- | --- | --- | --- | --- | --- | --- | --- |
|  | OR (95% CI) | OR (95% CI) | OR (95% CI) | OR (95% CI) | OR (95% CI) | OR (95% CI) | OR (95% CI) |
|  | N = 4485 | N = 4264 | N = 4088 | N = 3922 | N = 3574 | N = 3097 | N = 2960 |
| **Had asthma** |  |  |  |  |  |  |  |
| *No* |  |  |  |  |  |  |  |
| *Yes* | 3.17 (2.10-4.77)** | 4.09 (2.76-6.08)** | 3.29 (2.17-5.00)** | 3.66 (2.36-5.68)** | 3.66 (2.36-5.68)** | 3.17 (1.93-5.21)** | 1.99 (1.14-3.48)* |
| **Gestational age at birth** |  |  |  |  |  |  |  |
| *On time (37-41 weeks, ref.)* |  |  |  |  |  |  |  |
| *Early (36 weeks or less)* | 2.01 (1.07-3.76)* | 2.04 (1.19-3.52)* | 1.55 (0.91-2.64) | 0.67 (0.31-1.44) | 0.67 (0.31-1.44) | 0.81 (0.33-1.95) | 0.96 (0.36-2.53) |
| *Late (42 weeks or more)* | 1.18 (0.65-2.14) | 1.35 (0.72-2.53) | 1.30 (0.71-2.38) | 1.48 (0.74-2.94) | 1.48 (0.74-2.94) | 1.19 (0.56-2.56) | 2.07 (0.99-4.33)* |
| **Pre-pregnancy obesity** |  |  |  |  |  |  |  |
| *Healthy weight* |  |  |  |  |  |  |  |
| *Underweight* | 0.71 (0.39-1.28) | 0.74 (0.43-1.27) | 1.08 (0.66-1.76) | 0.89 (0.52-1.51) | 0.89 (0.52-1.51) | 1.38 (0.80-2.37) | 1.05 (0.56-1.94) |
| *Overweight* | 1.31 (0.90-1.91) | 1.04 (0.72-1.50) | 1.17 (0.81-1.67) | 1.21 (0.83-1.78) | 1.21 (0.83-1.78) | 1.47 (0.95-2.27)Ϯ | 1.08 (0.65-1.78) |
| *Obesity* | 1.56 (1.03-2.37)* | 1.35 (0.92-2.00) | 1.34 (0.90-2.00) | 1.12 (0.73-1.73) | 1.12 (0.73-1.73) | 0.88 (0.54-1.44) | 1.05 (0.63-1.74) |
| *Not known* | 1.67 (1.13-2.49)* | 1.30 (0.88-1.90) | 1.43 (0.97-2.11) | 1.74 (1.14-2.65)* | 1.74 (1.14-2.65)* | 1.12 (0.66-1.91) | 1.13 (0.66-1.93) |
| **Smoking during 1^st^ trimester** |  |  |  |  |  |  |  |
| *None (ref.)* |  |  |  |  |  |  |  |
| *<=10 cigarettes daily* | 0.93 (0.56-1.54) | 0.99 (0.60-1.63) | 0.90 (0.53-1.53) | 1.22 (0.67-2.24) | 1.22 (0.67-2.24) | 1.31 (0.69-2.47) | 1.01 (0.49-2.07) |
| *11+ cigarettes daily* | 1.54 (0.82-2.91) | 1.82 (0.96-3.46)Ϯ | 1.61 (0.84-3.06) | 2.07 (1.02-4.21)* | 2.07 (1.02-4.21)* | 1.56 (0.70-3.47) | 1.57 (0.63-3.90) |
|  |  |  |  |  |  |  |  |
| **Antibiotic medication** |  |  |  |  |  |  |  |
| *No* | 1.00 (0.62-1.6) | 1.02 (0.66-1.57) | 1.25 (0.81-1.94) | 1.39 (0.90-2.15) | 1.39 (0.90-2.15) | 1.08 (0.65-1.79) | 1.05 (0.62-1.77) |
| *Yes* |  |  |  |  |  |  |  |
| **Anti-depressant medication** |  |  |  |  |  |  |  |
| *No* | 0.75 (0.27-2.12) | 1.05 (0.44-2.52) | 1.65 (0.77-3.53) | 1.12 (0.38-3.31) | 1.12 (0.38-3.31) | 0.57 (0.13-2.47) | 0.82 (0.13-5.10) |
| *Yes* | 3.17 (2.10-4.77)** | 4.09 (2.76-6.08)** | 3.29 (2.17-5.00)** | 3.66 (2.36-5.68)** | 3.66 (2.36-5.68)** | 3.17 (1.93-5.21)** | 1.99 (1.14-3.48)* |

Notes: Ϯ: p<0.05 & >0.01; *: p<0.01 & >0.001; **: p<0.001; the regression models were adjusted for several covariates outlined in ‘Control variables’ sub-section of ‘Methods’ section.

**Table C6**: The risk of having ongoing asthma among female children based on the incidence of maternal asthma, other morbidities, and health behaviours during pregnancy

| **Maternal health, Risk factors, and medications during pregnancy** | **Age 2-3** | **Age 4-5** | **Age 6-7** | **Age 8-9** | **Age 10-11** | **Age 12-13** | **Age 14-15** |
| --- | --- | --- | --- | --- | --- | --- | --- |
|  | OR (95% CI) | OR (95% CI) | OR (95% CI) | OR (95% CI) | OR (95% CI) | OR (95% CI) | OR (95% CI) |
|  | N = 4485 | N = 4264 | N = 4088 | N = 3922 | N = 3574 | N = 3097 | N = 2960 |
| **Had asthma** |  |  |  |  |  |  |  |
| *No* |  |  |  |  |  |  |  |
| *Yes* | 2.08 (1.27-3.41)** | 2.68 (1.69-4.24)** | 3.40 (2.14-5.39)** | 2.83 (1.81-4.43)** | 4.09 (2.55-6.56)** | 3.38 (1.94-5.86)** | 3.30 (1.88-5.82)** |
| **Gestational age at birth** |  |  |  |  |  |  |  |
| *On time (37-41 weeks, ref.)* |  |  |  |  |  |  |  |
| *Early (36 weeks or less)* | 0.51 (0.20-1.31) | 0.47 (0.20-1.12)Ϯ | 0.41 (0.18-0.98)* | 0.49 (0.18-1.31) | 0.51 (0.17-1.60) | 0.42 (0.12-1.50) | 0.51 (0.16-1.60) |
| *Late (42 weeks or more)* | 0.30 (0.09-0.95)* | 0.68 (0.30-1.55) | 1.13 (0.52-2.49) | 0.72 (0.31-1.69) | 1.12 (0.49-2.59) | 1.09 (0.46-2.59) | 0.78 (0.33-1.87) |
| **Pre-pregnancy obesity** |  |  |  |  |  |  |  |
| *Healthy weight* |  |  |  |  |  |  |  |
| *Underweight* | 0.94 (0.52-1.71) | 0.66 (0.34-1.27) | 0.72 (0.39-1.35) | 1.23 (0.70-2.15) | 1.03 (0.54-1.99) | 0.55 (0.26-1.18) | 0.78 (0.39-1.57) |
| *Overweight* | 1.47 (0.96-2.26)Ϯ | 1.36 (0.92-2.02) | 1.34 (0.90-1.98) | 1.52 (1.02-2.28)* | 1.46 (0.94-2.27)Ϯ | 1.28 (0.80-2.05) | 1.21 (0.76-1.93) |
| *Obesity* | 1.51 (0.92-2.48) | 1.41 (0.91-2.19) | 1.80 (1.15-2.80)* | 1.79 (1.15-2.80)* | 1.53 (0.95-2.47)Ϯ | 2.00 (1.19-3.35)** | 1.69 (1.00-2.87)* |
| *Not known* | 1.05 (0.66-1.67) | 1.09 (0.71-1.69) | 1.53 (0.99-2.38)* | 1.66 (1.08-2.54)* | 1.85 (1.15-2.99) | 1.81 (1.10-2.98)* | 1.57 (0.95-2.58)Ϯ |
| **Smoking during 1^st^ trimester** |  |  |  |  |  |  |  |
| *None (ref.)* |  |  |  |  |  |  |  |
| *<=10 cigarettes daily* | 0.98 (0.53-1.84) | 0.87 (0.48-1.56) | 1.05 (0.56-1.98) | 1.26 (0.70-2.27) | 1.26 (0.64-2.46) | 1.15 (0.57-2.34) | 0.50 (0.2-1.27) |
| *11+ cigarettes daily* | 1.42 (0.73-2.77) | 1.94 (1.04-3.61) | 1.76 (0.91-3.43)Ϯ | 1.75 (0.85-3.60) | 1.51 (0.70-3.26) | 1.37 (0.62-3.04) | 1.62 (0.68-3.9) |
|  |  |  |  |  |  |  |  |
| **Antibiotic medication** |  |  |  |  |  |  |  |
| *No* | 1.55 (1.01-2.38)* | 1.25 (0.81-1.92) | 1.29 (0.84-1.96) | 0.92 (0.60-1.43) | 0.99 (0.61-1.59) | 0.77 (0.43-1.38) | 1.00 (0.55-1.81) |
| *Yes* |  |  |  |  |  |  |  |
| **Anti-depressant medication** |  |  |  |  |  |  |  |
| *No* | 0.98 (0.38-2.51) | 1.77 (0.69-4.55) | 1.33 (0.52-3.41) | 1.37 (0.52-3.59) | 2.19 (0.86-5.58) | 3.28 (1.23-8.71) | 1.13 (0.36-3.5) |
| *Yes* | 2.08 (1.27-3.41)** | 2.68 (1.69-4.24)** | 3.40 (2.14-5.39)** | 2.83 (1.81-4.43)** | 4.09 (2.55-6.56)** | 3.38 (1.94-5.86)** | 3.30 (1.88-5.82)** |

Notes: Ϯ: p<0.05 & >0.01; *: p<0.01 & >0.001; **: p<0.001; the regression models were adjusted for several covariates outlined in ‘Control variables’ sub-section of ‘Methods’ section.

**Table C7**: The risk of experiencing eczema among male children based on the incidence of maternal asthma, other morbidities, and health behaviours during pregnancy

| **Maternal health, Risk factors, and medications during pregnancy** | **Age 0-1** | **Age 2-3** | **Age 4-5** | **Age 6-7** | **Age 8-9** | **Age 10-11** | **Age 12-13** | **Age 14-15** |
| --- | --- | --- | --- | --- | --- | --- | --- | --- |
|  | OR (95% CI) | OR (95% CI) | OR (95% CI) | OR (95% CI) | OR (95% CI) | OR (95% CI) | OR (95% CI) | OR (95% CI) |
|  | N = 4977 | N = 4485 | N = 4264 | N = 4088 | N = 3922 | N = 3574 | N = 3097 | N = 2960 |
| **Had asthma** |  |  |  |  |  |  |  |  |
| *No* |  |  |  |  |  |  |  |  |
| *Yes* | 1.46 (0.96-2.21) | 1.13 (0.73-1.75) | 1.33 (0.83-2.13) | 2.08 (1.29-3.36)** | 1.76 (1.05-2.96)* | 2.19 (1.24-3.84)** | 1.16 (0.58-2.34) | 0.93 (0.41-2.09) |
| **Gestational age at birth** |  |  |  |  |  |  |  |  |
| *On time (37-41 weeks, ref.)* |  |  |  |  |  |  |  |  |
| *Early (36 weeks or less)* | 0.64 (0.36-1.13) | 0.98 (0.56-1.71) | 1.21 (0.67-2.19) | 1.39 (0.75-2.56) | 1.36 (0.68-2.73) | 0.70 (0.28-1.77) | 1.31 (0.51-3.35) | 0.85 (0.29-2.44) |
| *Late (42 weeks or more)* | 0.57 (0.30-1.09) | 0.65 (0.34-1.23) | 0.27 (0.09-0.78)* | 0.73 (0.34-1.58) | 0.97 (0.47-2.00) | 0.62 (0.25-1.57) | 0.53 (0.15-1.86) | 0.85 (0.31-2.35) |
| **Pre-pregnancy obesity** |  |  |  |  |  |  |  |  |
| *Healthy weight* |  |  |  |  |  |  |  |  |
| *Underweight* | 0.91 (0.59-1.39) | 0.53 (0.34-0.84)** | 0.44 (0.26-0.75)** | 0.69 (0.39-1.20) | 0.64 (0.34-1.21) | 0.97 (0.51-1.85) | 0.80 (0.39-1.65) | 1.09 (0.53-2.25) |
| *Overweight* | 1.21 (0.88-1.66) | 1.04 (0.76-1.41) | 0.95 (0.67-1.34) | 0.91 (0.62-1.36) | 1.15 (0.75-1.75) | 1.12 (0.70-1.79) | 0.84 (0.48-1.48) | 0.72 (0.37-1.41) |
| *Obesity* | 1.05 (0.72-1.51) | 1.09 (0.77-1.54) | 1.06 (0.71-1.58) | 1.05 (0.68-1.61) | 1.42 (0.90-2.24) | 0.94 (0.54-1.62) | 0.77 (0.42-1.41) | 1.6 (0.88-2.9) |
| *Not known* | 1.19 (0.85-1.65) | 0.92 (0.65-1.3) | 1.06 (0.72-1.56) | 0.97 (0.63-1.50) | 0.95 (0.59-1.53) | 1.10 (0.62-1.95) | 1.24 (0.67-2.31) | 1.02 (0.49-2.11) |
| **Smoking during 1^st^ trimester** |  |  |  |  |  |  |  |  |
| *None (ref.)* |  |  |  |  |  |  |  |  |
| *Occasional/<10 daily* | 1.16 (0.73-1.85) | 1.32 (0.87-2.01) | 1.29 (0.79-2.12) | 0.92 (0.50-1.70) | 0.67 (0.34-1.31) | 0.98 (0.45-2.14) | 0.70 (0.30-1.63) | 0.64 (0.22-1.82) |
| *11+ daily* | 0.54 (0.24-1.22) | 0.98 (0.51-1.88) | 0.84 (0.38-1.89) | 1.30 (0.55-3.06) | 0.51 (0.17-1.52) | 0.70 (0.17-2.86) | 1.17 (0.22-6.26) | 1.52 (0.47-4.97) |
| **Antibiotic medication** |  |  |  |  |  |  |  |  |
| *No* |  |  |  |  |  |  |  |  |
| *Yes* | 0.86 (0.58-1.28) | 1.01 (0.69-1.48) | 0.95 (0.62-1.44) | 0.98 (0.60-1.59) | 1.52 (0.95-2.42)Ϯ | 0.90 (0.50-1.62) | 1.77 (0.96-3.26)Ϯ | 1.38 (0.79-2.43) |
| **Anti-depressant medication** |  |  |  |  |  |  |  |  |
| *No* |  |  |  |  |  |  |  |  |
| *Yes* | 0.69 (0.28-1.71) | 1.84 (0.93-3.62)Ϯ | 1.28 (0.55-2.97) | 1.04 (0.42-2.56) | 0.37 (0.08-1.72) | 1.03 (0.30-3.48) | 1.99 (0.56-7.12) | 0.91 (0.11-7.82) |

Notes: Ϯ: p<0.05 & >0.01; *: p<0.01 & >0.001; **: p<0.001; the regression models were adjusted for several covariates outlined in ‘Control variables’ sub-section of ‘Methods’ section.

**Table C8**: The risk of experiencing eczema among female children based on the incidence of maternal asthma, other morbidities, and health behaviours during pregnancy

| **Maternal health, Risk factors, and medications during pregnancy** | **Age 0-1** | **Age 2-3** | **Age 4-5** | **Age 6-7** | **Age 8-9** | **Age 10-11** | **Age 12-13** | **Age 14-15** |
| --- | --- | --- | --- | --- | --- | --- | --- | --- |
|  | OR (95% CI) | OR (95% CI) | OR (95% CI) | OR (95% CI) | OR (95% CI) | OR (95% CI) | OR (95% CI) | OR (95% CI) |
|  | N = 4977 | N = 4485 | N = 4264 | N = 4088 | N = 3922 | N = 3574 | N = 3097 | N = 2960 |
| **Had asthma** |  |  |  |  |  |  |  |  |
| *No* |  |  |  |  |  |  |  |  |
| *Yes* | 1.46 (0.96-2.21) | 1.28 (0.82-2.01) | 1.62 (0.99-2.66)* | 2.08 (1.29-3.36)* | 1.11 (0.67-1.85) | 1.03 (0.58-1.83) | 2.15 (1.21-3.83) | 1.95 (1.08-3.51)* |
| **Gestational age at birth** |  |  |  |  |  |  |  |  |
| *On time (37-41 weeks, ref.)* |  |  |  |  |  |  |  |  |
| *Early (36 weeks or less)* | 0.64 (0.36-1.13) | 0.70 (0.38-1.30) | 0.61 (0.28-1.32) | 1.39 (0.75-2.56) | 0.47 (0.16-1.36) | 0.68 (0.22-2.13) | 0.24 (0.07-0.78)Ϯ | 0.60 (0.17-2.18) |
| *Late (42 weeks or more)* | 0.57 (0.30-1.09) | 0.60 (0.30-1.21) | 0.75 (0.36-1.56) | 0.73 (0.34-1.58) | 0.89 (0.44-1.82) | 0.62 (0.29-1.33) | 0.52 (0.18-1.52) | 1.03 (0.43-2.45) |
| **Pre-pregnancy obesity** |  |  |  |  |  |  |  |  |
| *Healthy weight* |  |  |  |  |  |  |  |  |
| *Underweight* | 0.91 (0.59-1.39) | 0.86 (0.54-1.37) | 1.07 (0.64-1.77) | 0.69 (0.39-1.20) | 0.79 (0.45-1.4) | 1.37 (0.75-2.5) | 1.07 (0.51-2.22) | 0.62 (0.30-1.29) |
| *Overweight* | 1.21 (0.88-1.66) | 0.97 (0.70-1.35) | 1.19 (0.82-1.73) | 0.91 (0.62-1.36) | 1.18 (0.82-1.71) | 1.52 (1.00-2.31)* | 1.81 (1.13-2.93) | 1.10 (0.68-1.78) |
| *Obesity* | 1.05 (0.72-1.51) | 1.23 (0.85-1.77) | 1.39 (0.93-2.09) | 1.05 (0.68-1.61)* | 1.19 (0.77-1.82) | 1.62 (0.99-2.63)* | 1.80 (1.00-3.24) | 2.03 (1.22-3.36)** |
| *Not known* | 1.19 (0.85-1.65) | 0.95 (0.67-1.36) | 1.42 (0.95-2.14) | 0.97 (0.63-1.50) | 0.97 (0.61-1.54) | 1.16 (0.69-1.97) | 1.85 (1.05-3.26) | 1.09 (0.60-1.96) |
| **Smoking during 1^st^ trimester** |  |  |  |  |  |  |  |  |
| *None (ref.)* |  |  |  |  |  |  |  |  |
| *Occasional/<10 daily* | 1.16 (0.73-1.85) | 1.02 (0.61-1.69) | 0.97 (0.55-1.71) | 0.92 (0.50-1.70) | 0.96 (0.52-1.76) | 0.65 (0.31-1.35) | 0.50 (0.18-1.43) | 0.49 (0.18-1.36) |
| *11+ daily* | 0.54 (0.24-1.22) | 0.87 (0.44-1.73) | 0.61 (0.29-1.30) | 1.30 (0.55-3.06) | 1.18 (0.52-2.67) | 0.75 (0.28-1.98) | 1.07 (0.40-2.90) | 0.88 (0.34-2.24) |
| **Antibiotic medication** |  |  |  |  |  |  |  |  |
| *No* |  |  |  |  |  |  |  |  |
| *Yes* | 0.86 (0.58-1.28) | 1.04 (0.70-1.53) | 1.08 (0.71-1.64) | 0.98 (0.60-1.59)* | 1.13 (0.73-1.75) | 1.47 (0.92-2.34) | 0.76 (0.38-1.55) | 1.31 (0.72-2.38) |
| **Anti-depressant medication** |  |  |  |  |  |  |  |  |
| *No* |  |  |  |  |  |  |  |  |
| *Yes* | 0.69 (0.28-1.71) | 1.47 (0.59-3.67) | 1.37 (0.58-3.22) | 1.04 (0.42-2.56) | 1.7 (0.71-4.1) | 0.94 (0.33-2.71) | 0.40 (0.08-2.03)Ϯ | 0.40 (0.09-1.78) |

Notes: Ϯ: p<0.05 & >0.01; *: p<0.01 & >0.001; **: p<0.001; the regression models were adjusted for several covariates outlined in ‘Control variables’ sub-section of ‘Methods’ section.
